# Long-term morphological and functional dynamics of human stem cell-derived neuronal networks on high-density micro-electrode arrays

**DOI:** 10.3389/fnins.2022.951964

**Published:** 2022-10-04

**Authors:** Rouhollah Habibey, Johannes Striebel, Felix Schmieder, Jürgen Czarske, Volker Busskamp

**Affiliations:** ^1^Department of Ophthalmology, Universitäts-Augenklinik Bonn, University of Bonn, Bonn, Germany; ^2^Laboratory of Measurement and Sensor System Technique, Faculty of Electrical and Computer Engineering, TU Dresden, Dresden, Germany; ^3^Competence Center for Biomedical Computational Laser Systems (BIOLAS), TU Dresden, Dresden, Germany; ^4^Cluster of Excellence Physics of Life, TU Dresden, Dresden, Germany; ^5^School of Science, Institute of Applied Physics, TU Dresden, Dresden, Germany

**Keywords:** electrophysiology, burst activity, GABAergic synapse, high-density micro-electrode array, human stem cell-derived neurons, long-term culture, network development, network morphology

## Abstract

Comprehensive electrophysiological characterizations of human induced pluripotent stem cell (hiPSC)-derived neuronal networks are essential to determine to what extent these *in vitro* models recapitulate the functional features of *in vivo* neuronal circuits. High-density micro-electrode arrays (HD-MEAs) offer non-invasive recording with the best spatial and temporal resolution possible to date. For 3 months, we tracked the morphology and activity features of developing networks derived from a transgenic hiPSC line in which neurogenesis is inducible by neurogenic transcription factor overexpression. Our morphological data revealed large-scale structural changes from homogeneously distributed neurons in the first month to the formation of neuronal clusters over time. This led to a constant shift in position of neuronal cells and clusters on HD-MEAs and corresponding changes in spatial distribution of the network activity maps. Network activity appeared as scarce action potentials (APs), evolved as local bursts with longer duration and changed to network-wide synchronized bursts with higher frequencies but shorter duration over time, resembling the emerging burst features found in the developing human brain. Instantaneous firing rate data indicated that the fraction of fast spiking neurons (150–600 Hz) increases sharply after 63 days post induction (dpi). Inhibition of glutamatergic synapses erased burst features from network activity profiles and confirmed the presence of mature excitatory neurotransmission. The application of GABAergic receptor antagonists profoundly changed the bursting profile of the network at 120 dpi. This indicated a GABAergic switch from excitatory to inhibitory neurotransmission during circuit development and maturation. Our results suggested that an emerging GABAergic system at older culture ages is involved in regulating spontaneous network bursts. In conclusion, our data showed that long-term and continuous microscopy and electrophysiology readouts are crucial for a meaningful characterization of morphological and functional maturation in stem cell-derived human networks. Most importantly, assessing the level and duration of functional maturation is key to subject these human neuronal circuits on HD-MEAs for basic and biomedical applications.

## Introduction

Intricate brain networks are derived from sophisticated but organized connections between neuronal cells that evolve during brain development (Bassett and Sporns, 2017; Kriegeskorte and Douglas, 2018). Structural and functional remodeling of brain circuits are driven by a complex interplay of genetic and epigenetic programs (Tau and Peterson, 2010). These changes in the shape and activity emerge naturally during a predictable developmental timeline (Stiles and Jernigan, 2010; Gozdas et al., 2019; Martinez and Sprecher, 2020; Kim and Paredes, 2021). One of the challenging scientific endeavors in neuroscience is to decipher the process of functional development and network organization (Sporns, 2013; Rosenthal et al., 2018; van Atteveldt et al., 2021). In this regard, studies on human and animal models have extensively explored neuronal networks, providing substantial understanding of their self-assembling and their function *in vivo* (Tau and Peterson, 2010; Bassett and Gazzaniga, 2011; Latifi et al., 2020; Huber et al., 2021; Levakov et al., 2021; Li et al., 2021).

Neurodevelopmental research in humans is mostly limited to *postmortem* and neuroimaging techniques, hindering detailed developmental studies using electrophysiology tools (Manzini et al., 2021; Mezinska et al., 2021). The human brain develops through proliferation and differentiation of neuronal progenitor cells, migration of immature neurons, axonal elongation and pathfinding, dendrite growth and arborization and synaptogenesis (Raybaud et al., 2013; Budday et al., 2015; Jiang and Nardelli, 2016; Silbereis et al., 2016). This leads to an overconnectivity phase that is followed by a critical period of synapse pruning to establish organized brain circuits (Craig et al., 2006; Craik and Bialystok, 2006; Budday et al., 2015; Silbereis et al., 2016). In mammals, excitatory neurons mature at earlier embryonic stages while GABAergic neurons develop later (Warm et al., 2022). Structural and functional maturation of excitatory and inhibitory circuits emerge as distinct activity patterns during cortex development (Luhmann et al., 2016; Teppola et al., 2019). Network-wide synchronized burst activity attributed to glutamatergic synapses appears midterm, develops until birth and then decreases by maturation of the GABAergic system (Corlew et al., 2004; Kirwan et al., 2015). The GABAergic system first emerges as neurons with excitatory synaptic transmission, however, upon maturation in the early postnatal period it switches to inhibitory neurotransmission (Ganguly et al., 2001; Zafeiriou et al., 2020).

As an alternative approach, *in vitro* neuronal culture systems are used to scale down network complexity to study functional features of developing neuronal networks under precise experimental conditions (Pastrana, 2013; Hattori, 2014; Paşca, 2018; Ndyabawe and Kisaalita, 2019). Human stem cell derived neuronal networks offer the potential to recapitulate developmental features of brain circuits *in vitro* (Odawara et al., 2014; Sasaki et al., 2019; Schmieder et al., 2020, 2022; Ronchi et al., 2021). Rapid progress in human-derived stem cell technology facilitated the generation of *in vitro* culture systems as an important complementary platform to animal models (Ardhanareeswaran et al., 2017). From a biomedical perspective, human-derived *in vitro* models offer the potential to reduce the translational gap from bench-to-bedside by allowing an early incorporation of human disease models bearing disease-causing mutations (Nikolakopoulou et al., 2021). Two- or three-dimensional human neuronal cultures are largely engineered using neuronal cells derived from human embryonic (hESCs) (Ilic and Ogilvie, 2017) or human induced pluripotent stem cells (hiPSCs) (Hockemeyer and Jaenisch, 2016; Shi et al., 2017). Multiple steps of differentiating stem cells into postmitotic neurons often demand weeks or months of cell culture (Zhang et al., 2013; Canals et al., 2018). This has been simplified by single-step induction of neurogenic transcription factor expression (Pang et al., 2011; Zhang et al., 2013; Busskamp et al., 2014). An example of a well-established transgenic hiPSC is the inducible neurogenin (iNGN) cell line, in which the overexpression of neurogenin-1 and neurogenin-2 can be induced by the TetOn promoter system (Busskamp et al., 2014; Lam et al., 2017; Kutsche et al., 2018; Ng et al., 2021). These cells are differentiated into postmitotic neurons within 4 days post induction (dpi) (Busskamp et al., 2014) but require additional time for functional maturation subsequently (Lam et al., 2017; Schmieder et al., 2022).

Human-derived neuronal networks require comprehensive and long-term electrophysiological validation and characterization to serve as a reliable platform for addressing biomedical questions or modeling neurodevelopment *in vitro* (Mossink et al., 2021; Habibey et al., 2022; Schmieder et al., 2022). Patch-clamp recordings have been used to characterize the functional properties of iNGN-derived neurons at different time points over 100 dpi (Lam et al., 2017). However, in order to study the same neuronal circuit over months it is necessary to record from multiple neurons simultaneously without damaging or contaminating them (Habibey et al., 2020, 2022). Standard micro-electrode arrays (MEAs) are used to non-invasively measure activity from networks of primary neurons (Habibey et al., 2015a,^2017^; Latifi et al., 2016), stem-cell derived neuronal networks (Odawara et al., 2014; Habibey et al., 2022; Schmieder et al., 2022), and neuronal tissue slices (Steidl et al., 2006; Mannal et al., 2021) but offer limited spatial resolution of network activity. High-density MEAs (HD-MEAs) are based on complementary-metal-oxide-semiconductor (CMOS) technology (Ballini et al., 2014) and became available during the past decade. These devices significantly improve the spatial resolution of electrophysiology readout and enable single neuron and sub-cellular recordings (Berdondini et al., 2009; Ballini et al., 2014; Müller et al., 2015). Standard and HD-MEAs advanced electrophysiological research and are a suitable tool for long-term investigation of developing neuronal networks (Berdondini et al., 2009; Ballini et al., 2014; Müller et al., 2015; Habibey et al., 2022). They also have been used to characterize the electrophysiological properties of primary or iPSCs-derived neuronal circuits (Amin et al., 2016; Matsuda et al., 2018; Ronchi et al., 2021).

Cultured hiPSC-derived neuronal networks on HD-MEAs have shown an increase in neuronal firing rates and synchronized burst activity by culture age (Lu et al., 2019). These basic electrophysiological features have been extracted from networks of different cell lines, e.g., diseased cell lines, to reveal their functional phenotype (Ronchi et al., 2021). Such data are of great value to investigate self-assembling neuronal networks *in vitro* over time. This is especially useful for studying the functional features of such networks (Akarca et al., 2022) and their capability of recapitulating developing brain circuits. To date, there are only few studies that have aimed to characterize long-term functional features from developing primary (Habibey et al., 2015a,b) and stem cell-derived neuronal networks (Odawara et al., 2014, 2016; Amin et al., 2016; Lu et al., 2019). In this study we investigated long-term activity features of iNGN-EGFP-derived neuronal networks using HD-MEAs and revealed large-scale changes of their network morphology over time. We discovered wide-range shifts in neuronal cell position and network morphological transformation from homogenously distributed single neurons in earlier days to robustly clustered networks after 3 months. We found dynamic network location and activity shifts of neuronal cells and clusters on the sensor area (network activity image). Our data suggest that structure-dependent shifts in the network activity should be taken into consideration when measuring the functional phenotype of the developing networks *in vitro*. We revealed emerging and evolving features of burst activity comparable to the development of network bursts in the pre- and postnatal human brain.

## Materials and methods

### Long-term neuronal culture on high-density micro-electrode arrays

**FIGURE 1:**
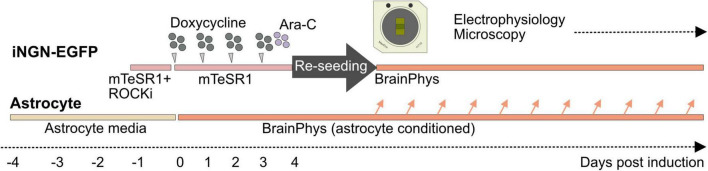
Seeding iNGN-EGFP cells in two step and supporting them with astrocyte-conditioned medium. iNGN-EGFP cells were differentiated for 4 days on a Matrigel coated plate. Ara-C was applied on 3 dpi to remove undifferentiated cells. On 4 dpi iNGN-EGFP cells were dissociated and seeded on a PDL-laminin coated HD-MEA chip. Parallel astrocyte culture on PDL-laminin coated glass coverslips was used to provide astrocyte-conditioned medium for neuronal cultures.

A two-step cell-seeding protocol that has been developed for standard low-density MEA devices (Habibey et al., 2022) was adapted to generate long-term neuronal cultures within HD-MEA chips (Figure 1). In the first step, iNGN-EGFP cells were differentiated to neurons and then neurons were detached and reseeded on PDL-laminin coated MEA devices.

Preparation of HD-MEA chips and coating with PDL-laminin: Sterile HD-MEA chips (MaxOne, MaxWell Biosystems, Switzerland) were plasma treated for 2 min (Diener Electronics, Germany; ambient air, 0.3 mbar) to improve the surface hydrophilicity. A PDL (0.1 mg/ml, 200 μl, Merck) coating was added (50 μl) to cover the sensor area and its surrounding regions inside the chip. The PDL coating was incubated for 24 h, then HD-MEA chips were washed with ultrapure water (3x) and dried under laminar flow. Laminin (50 μl, 0.05 mg/ml concentration, Sigma, Germany) was added to the sensor area and incubated for 3 h directly before reseeding.

Differentiation of iNGN-EGFP cells to neurons on Matrigel-coated plates: Human-derived iNGN-EGFP cells (Busskamp et al., 2014) were differentiated for 4 days in a Matrigel (Corning)-coated 6-well plate. For 4 days differentiation, iNGN-EGFP cells were kept in mTeSR1™ medium (mTeSR1™) Basal Medium with mTeSR1™ 5 × Supplement (Stemcell Technologies, Germany) and 1% penicillin-streptomycin (P/S; Thermo Fisher Scientific, Germany). The mTeSR1™ media was exchanged every day. For neuronal induction, iNGN-EGFP cells were treated with 0.5 μg/ml doxycycline per day (Sigma, Germany) for 4 consecutive days (Figure 1). Ara-C (5 μM; cytosine β-D-arabinofuranoside hydrochloride, Sigma, Germany) was applied at 3 dpi to remove undifferentiated cells before re-seeding.

Reseeding differentiated neurons on HD-MEA devices: Differentiated iNGN-EGFP neurons were dissociated by Accutase (Sigma, Germany), centrifuged at 359 g for 4 min, and after removing the supernatant, the cell pallet was resuspended in complete BrainPhys™ [BrainPhys™ Neuronal Medium (Stemcell Technologies, Germany) + 1% P/S + NeuroCult™ SM1 Neuronal Supplement (Stemcell Technologies, Germany) + N2 Supplement-A + 20 ng/ml recombinant human BDNF (Peprotech, Germany) + 20 ng/ml recombinant human GDNF (Peprotech, Germany) and 200 nM ascorbic acid (Sigma, Germany)]. Cells were reseeded on the sensor area (around 30,000 cells per sensor area).

Supporting long-term cultures with astrocyte-conditioned media: To support neurons with astrocyte-secreted factors, we prepared rat primary astrocyte cultures (Thermo Fisher Scientific, A1261301, Germany) 4 days before neuronal induction (Figure 1). Astrocytes were seeded on PDL (0.1 mg/ml), and laminin (0.05 mg/ml) coated 18 mm coverslips. Astrocytes were kept in astrocyte medium (DMEM + 4.5 g/l d-glucose + pyruvate plus N2 Supplement, 10% One Shot™ fetal bovine serum and 1% P/S (all provided by Thermo Fisher Scientific, Germany). Four days before reseeding, the astrocyte medium was drained, cells were washed with warm PBS without calcium and magnesium (Thermo Fisher Scientific, Germany) and fresh complete BrainPhys medium was added. Astrocyte-conditioned complete BrainPhys™ media was mixed with fresh complete BrainPhys™ (1:3) to replace half of the medium in HD-MEAs once every week.

### Microscopy

**FIGURE 2:**
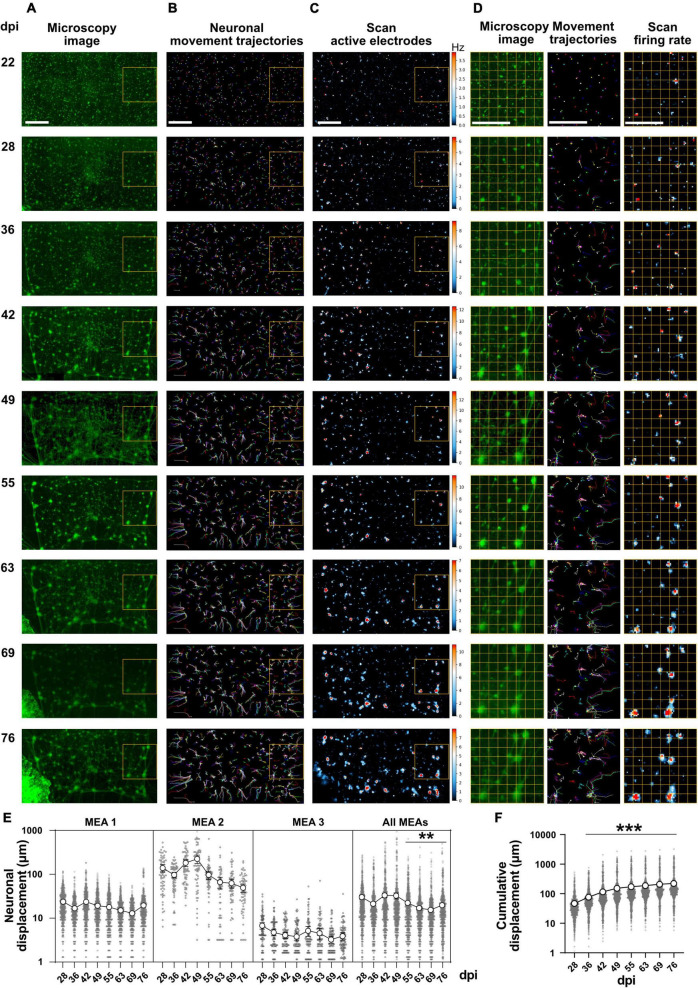
Network morphology and activity images at different days post induction. **(A)** Fluorescence microscopy images from whole networks were prepared by stitching 40 images at each time point. Magnified view of network morphology on 22, 28, and 63 dpi are illustrated in Supplementary Figure 1. Scale bar is 500 μm. **(B)** Neuronal movement trajectories were extracted from morphology image sequences at different weeks. A magnified view of the trajectories is shown in Supplementary Figure 2. Scale bar is 500 μm. **(C)** Network activity image based on firing rates. Each day, a full Activity Scan was performed that included 30 s recording from each electrode. Electrodes that showed activity are represented by blue color. The color code indicates firing rates from no activity as black, to medium activity as blue and maximum firing rates as red (firing rates have not been normalized across different days and color coding represents different scales of the activity between frames). Scale bar is 500 μm. **(D)** Magnified view of a network region (left column), neuronal movement trajectory (middle) and activity image of the same region (right column) at different days post induction (marked by yellow rectangles in **A–C**). Each image in **(D)** represents a sensor area including approximately 56 × 56 electrodes and each small square with yellow border represents a sensor area of approximately 7 × 7 electrodes. Scale bar is 500 μm. **(E)** Neuronal displacement at different days during network development in 3 individual HD-MEAs with 721, 68, and 115 neurons tracked in each MEA, respectively, and all MEAs together (*N* = 3 MEAs). Each circle represents the distance that a neuron shifted from the previous time point (dpi). Average displacement (*n* = 904 neurons) is represented as big circles with lines (**p < 0.01 vs. 28 dpi. **F**). Cumulative displacement of neuronal soma position between 22 and 76 dpi for 904 neurons of 3 HD-MEAs (***p < 0.001 vs. 28 dpi). Data have been collected from 904 neurons in 3 HD-MEAs at 8 time points between 28 and 76 dpi and were compared using Kruskal-Wallis test followed by Dunn’s multiple comparison test. Percentage of neurons showed specific measure of displacement have been represented in Supplementary Figure 4.

Microscopy images from each culture were prepared at different days using an upright fluorescent microscope (Olympus SliceScope Pro 6000, Scientifica, UK). Cultures were placed on a temperature controller top plate (Tokai Hit, Inu-Kiw-F1, Japan) during imaging. A custom made thin and transparent cell culture lid was fabricated from Polydimethylsiloxane (PDMS) to prevent evaporation and allow upright microscopy of the samples in sterile conditions. For fluorescent imaging, a pE-300ultra system (CoolLED, UK) was used to generate blue excitation light. It was guided through a liquid light guide and a pE-Universal Collimator (CoolLED, UK) to the epifluorescence port of the microscope. A GFP filter set (SCI-49002s, Scientifica, UK), including 470/40x, 495LP, 525/50 m filters, was used for fluorescent imaging of the sample. Emitted light was detected by a CCD camera (SciCam Pro, 100918 Scientifica, UK) and processed in Ocular software (Scientifica, UK). To image the whole network on the sensing area, 40 mosaic images were prepared from each network (10x, OLY-N1215800 objective). These images were later stitched together using the ImageJ software and the Grid/Collection stitching plugin (Preibisch et al., 2009) to visualize the overall network morphology at each specific day post induction. Morphology images prepared from three HD-MEA cultures in 9 subsequent weeks from 22 to 76 dpi were used to generate time-lapse videos to show changes in network structure (Figure 2 and Supplementary Figure 1). A manual tracking plugin was used for tracking the movement trajectories of the neuronal cells and clusters over 2 months. Sequences of network images at different days were imported into ImageJ and neurons were selected and tracked individually in 3 MEAs. For neurons that joined clusters we tracked the clusters. Movement trajectories of all tracked neurons were saved as images and video files (Figure 2 and Supplementary Figures 2, 3) and AVI format. Neuronal cell and cluster displacement data (*N* = 3 MEAs and *n* = 904 neurons) on the sensor area were extracted as CSV file and used to measure the movement per week or overall displacement during the months.

### Electrophysiology recordings

Single-well HD-MEAs (MaxOne, MaxWell Biosystems, Switzerland) were used for plating neurons and recording from developing networks. MaxOne MEAs consist of 26,400 electrodes that have been arranged in a 120 × 220 configuration with 17.5 μm center-to-center electrode pitch (Ballini et al., 2014; Müller et al., 2015). These electrodes cover a large sensor area (3.85 × 2.10 mm^2^). A MaxOne HD-MEA system (MaxWell Biosystems, Switzerland) was used to readout electrophysiology data from MaxOne chips. The MaxOne HD-MEA system was placed in a digital mini-incubator (VWR, Germany) to provide stable temperature conditions (37°C) during the recording. For all recordings we used the MaxLab Live software (MaxWell Biosystems, Switzerland). In all recording sessions, first, an Activity Scan Assay was performed in full scan mode to extract the active electrodes and activity image (or activity map) of the active electrodes based on their firing rate. Activity scans in the full scan mode provides the highest possible scanning solution. To this end, scanning starts automatically by recording from around 1,000 electrodes for a selected time period (e.g., 30 s) and then moves to the next set of electrodes. This process iterates 29 times to capture activity of electrodes over the entire sensor area. Based on scan results, the software calculates the activity parameters (e.g., firing rate and spike amplitude) for each electrode. Electrodes that showed more than 0.1 Hz AP frequency and average AP amplitude more than 20 μV were considered as active electrodes. A color-coded map of the whole sensor area indicating the activity level in each electrode was created (activity image/activity map). Then, we selected up to 1,024 of the most active electrodes and ran a Network Assay to simultaneously record from these electrodes for 5 min. Recorded files were used for activity analysis, burst analysis, and measurement of instantaneous firing rate. Network activity data of three HD-MEA cultures were recorded on 11 subsequent weeks between 22 and 90 dpi for measuring long-term changes in activity and burst features. Overall, the activity scan and follow up simultaneous recordings from selected neurons required around 45 min. To limit the recorded file size and recording time we had to limit the scanning time for each electrode to 30 s (which has been also proposed by the vendor). As an inevitable drawback of this method if an electrode was not active during that short period of scanning time it was excluded from analysis. The experiment was started with six HD-MEA chips of which three HD-MEAs showed stable network structure and activity for more than 3 months. The networks of the excluded MEAs have been detached in the first 3 weeks of culture and were excluded at 35 dpi.

Application of glutamatergic and GABAergic synapse blockers: Two HD-MEA cultures were used to test the inhibition of excitatory glutamate receptors and inhibitory GABAergic receptors on 118 and 120 dpi, respectively. To block glutamatergic receptors (AMPA receptors: The α-amino-3-hydroxy-5-methyl-4-isoxazolepropionic acid receptors, and NMDA receptors: N-methyl-D-aspartate receptors), a combination of 2,3-dihydroxy-6-nitro-7-sulfamoyl-benzo[f]quinoxaline (NBQX; an AMPA receptor antagonist) and (2R)-amino-5-phosphonopentanoate (APV; a selective NMDA receptor antagonist) were applied to each culture (Schmieder et al., 2022). First, 500 μl of the culture medium in each HD-MEA was drained and kept for later use. Then, Activity Scan and Network Assay were performed to record spontaneous network activity before applying NBQX+APV. Thereafter, NBQX (10 μM) and APV (50 μM) were added to each HD-MEA culture and incubated for 5 min in 37°C. Activity Scan and Network Assay were performed in the presence of NBQX+APV. The medium inside the HD-MEA was drained and cells were washed twice with warm complete BrainPhys™ medium. Pre-used medium was mixed with fresh BrainPhys™ medium (1:1), added to the HD-MEA culture and incubated for 30 min. Activity Scan and Network Assay were performed again after washout. The same steps of experiment and recording were applied for GABA-A receptor inhibition by Gabazine (10 μM) on 120 dpi.

### Spike sorting

To identify individual neuronal units from extracellular recordings, all signal traces of the electrodes were spike sorted. Spike sorting was performed on the 5 min Network Assay data. All sorting steps including the pre- and postprocessing of the data were performed with the SpikeInterface framework (Buccino et al., 2020) and the Kilosort3 algorithm (Pachitariu et al., 2016). Preprocessing of the data consisted of applying a 2^nd^ order Butterworth high pass filter with a cutoff frequency of 100 Hz and a common mean referencing step. Sorting was performed with default parameters, except that batch size was decreased to NT = 16,448 to avoid memory issues. The processed output of the spike sorting was automatically curated and units satisfying at least one the following conditions were removed: interspike interval (ISI) violation rate above 0.2, signal to noise ratio smaller than 5 and firing rate below 0.1 Hz.

### Action potential frequency, instantaneous firing frequency and burst features

Extracted time stamps from sorted data were imported into NeuroExplorer software (version 5) for analysis. AP and burst parameters were measured in 3 HD-MEA chips at 11 different time points from 22 to 90 dpi. AP frequency is calculated by dividing the total number of recorded APs by recording duration in seconds. Instantaneous firing rates capture the firing frequency of individual APs based on their interspike intervals (ISI). It is calculated by taking the inverse value of the ISI using instant frequency algorithm in Neuroexplorer (Wang et al., 2016). Instantaneous frequency was used to calculate following parameters: average instantaneous frequency in each day, percentage of fast firing APs (or spiking events) during the 5 min recording time period, and percentage of neurons that showed fast firing rates. Later we categorize neurons as fast firing neurons (>150 Hz) and slow firing neurons (<150 Hz). To measure the burst activity features we checked available algorithms in Neuroexplorer for detecting the burst events in neuronal data including an interval specification algorithm, a firing-rate-based algorithm and a surprise (probability-based) algorithm. Based on visual inspections, the surprise algorithm worked best on our dataset. The interval specification method included false positive bursts, and the firing rate-based method excluded most of the burst features. Bursts were detected and extracted using the surprise (probability) algorithm with the following parameters: 4 as minimum surprise, 20 ms minimum duration of the burst and 4 as minimum number of APs in the burst (Habibey et al., 2015b).

The Poisson surprise (PS) for a given time T containing *N* action potentials (APs) is calculated as:

S=-log⁡p

With,

$$P=\exp\left(-\lambda \,T\,\sum_{n=N}^{\infty }\frac{{\left(-\lambda \,T\right)}^{n}}{n!}\right)$$

and λ being the mean AP firing rate. Considering that P is the probability of *N* or more APs occurring randomly in a time period of *T*. NeuroExplorer uses a surprise maximization algorithm (Legendy and Salcman, 1985; Cotterill and Eglen, 2019) to find bursts across the AP trains of a single neuron based on following protocol: A burst is identified if three consecutive APs have an ISI of less than half of the average ISI of the spike train. Subsequent APs are added to the initial burst until the ISI becomes larger than the average ISI. For each added AP, the surprise is calculated and at the end the burst length with maximum surprise is selected. Then scanning is moving forward to find another burst.

Burst features including burst frequency, burst duration, and percentage of APs in the burst events were extracted and used for tracking the network activity dynamics during development (Wilk et al., 2016; Habibey et al., 2017). The percentage of APs in burst represents which percentage of overall APs in a neuron happens inside the burst events.

### Statistical analysis

The number of active neurons within each HD-MEA device and in each specific culture age was determined based on their AP frequencies. Neurons with more than 0.1 Hz firing rate were considered as active neurons and were included. Data was collected from three HD-MEAs with a minimum of 1,065 sorted neurons in total on 22 dpi to a maximum of 3,076 detected neurons on 69 dpi. The number of sorted neurons at all-time points ranged between these two values. The average AP frequency between different days was compared using the Kruskal-Wallis test followed by Dunn’s multiple comparison test. The same test was applied to compare burst features, instantaneous frequency and neuronal movement between different days. All data are presented as mean ± standard error of mean and *p*-values less than 0.05 were considered significant. We also applied the Kruskal-Wallis test followed by Dunn’s multiple comparison test to compare baseline and washout conditions with NBQX+APV or gabazine treated conditions on 118 and 120 dpi, respectively.

## Results

### Network morphology and activity images show correlated long-term changes

HD-MEAs allow the scanning of all available sensing electrodes to extract a map of active electrodes with their firing rates. We used this advantage to find out how changes of the network structure over time are reflected in changes or shifts of the activity image of the network. Starting from 22 dpi, microscopy images of the whole sensor area (*n* = 3) were prepared and compared to maps created from Activity Scans (Figure 2). Microscopy images showed that homogenously distributed single neurons across the sensing area tend to cluster together (Supplementary Figure 1). These small clusters (diameter < 50 μm) formed around 30 dpi and are connected through axonal bundles to other clusters (Figure 2 and Supplementary Video 1). Manual tracking of neuronal cell body also confirmed clusterization of the neurons (Supplementary Video 2). As the cultures became older, some of the small clusters merged and formed large clusters (diameter > 100 μm, Figure 2, Supplementary Figure 1, and Supplementary Videos 1, 2). Time lapse images of cultures showed that this mechanical interaction between neurons and clusters constantly shifted their position on the surface of the sensing area (Supplementary Video 1). The distance that a neuron travels in each week or during the whole period of the experiment was calculated for 904 neurons in 3 HD-MEAs based on neuronal tracking data (Figure 2 and Supplementary Video 2). These data showed that at earlier time points and before cluster formation, neuronal displacement was higher compared to later time points (28.04 ± 1.40 μm per week on 28 dpi vs. 19.14 ± 0.75 μm per week on 76 dpi, *p* < 0.01; Figure 2E). The average displacement of neurons in each chip varied between 35.95 ± 1.96 and 1,027 ± 52.20 μm (Figure 2E). The cumulative displacement of neurons from their original position at 22 dpi showed an average shift of 224.00 ± 10.10 μm after 2 months (Figure 2F). Among all 904 tracked neurons of 3 HD-MEA cultures 76.01% shifted between 50 and 200 μm in 2 months (Supplementary Figure 4). Considering the 17 μm electrode pitch and average displacement of neurons around 224 μm, the center of mass of the activity of a neuron is shifting by more than 12 different electrodes on average. Since one neuron is recorded by several electrodes already without movement, the overall number of electrodes capturing its activity is likely much higher. Extracted activity maps from the same networks over time showed that the active regions were also regularly shifting across the sensor (Figure 2C and Supplementary Video 3). Magnified images and time laps videos revealed that alterations in shape and position of the activity maps were tightly correlated with movements of neurons and neuronal cluster on the sensor surface (Figure 2D and Supplementary Video 4). These data indicated that each network cluster is detected by a different set of electrodes as the network develops over weeks and months. Similar changes in network morphology were observed on standard MEAs with limited number of electrodes in our previous work (Supplementary Video 5; Schmieder et al., 2022).

### Long-term development of spontaneous activity and burst features in inducible neurogenin-derived neuronal networks

**FIGURE 3:**
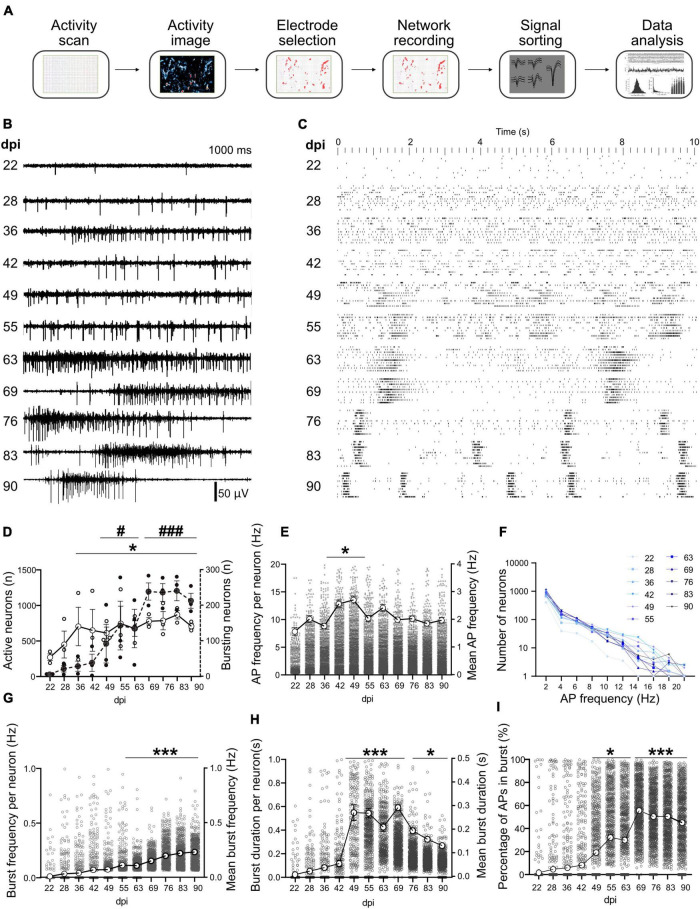
Long-term activity profile of developing hiPSCs-derived networks. **(A)** Pipeline of data acquisition and analysis. After a full activity scan of the whole array and extracting the activity image, the most active electrodes were selected and used for simultaneous recording for network analysis. Recorded data was spike sorted to identify neurons, then timestamps of neuronal activity were extracted and used for data analysis. **(B)** Representative signal trace in a selected electrode at different days post induction (dpi). **(C)** Ten second raster plot profile of detected APs in 10 neurons (aligned in rows) on different dpi. **(D)** Number of active neurons and number of neurons involved in network burst activity. Neurons that showed more than 0.1 Hz AP frequency were considered as active neurons. **(E)** Average AP frequency was calculated by dividing the total number of APs to the overall recording period at each dpi. **(F)** Number of neurons firing in different frequency domains at different days (color-coded). **(G–I)** Burst frequency, burst duration and percentage of APs that appear inside burst events at different days. Data of *N* = 3 MEAs with *n* > 1,065 neurons per day were included in the analysis **(D–I)** and compared between days based on Kruskal-Wallis test followed by Dunn’s multiple comparison test. *p < 0.05, and ***p < 0.001 vs. 22 and 28 dpi. For the number of neurons that contributed to burst activity #*p* < 0.05 and ###*p* < 0.001 vs. 22, 28, and 36 dpi. Each point represents the corresponding activity feature in one neuron.

Data extracted (Figure 3A) from 3 MEAs at different dpi were used for tracking the network functional features. Activity appeared as individual APs in electrodes and corresponding neurons earlier than 22 dpi (Figure 3B). This changed to clear network burst activity around 63 dpi (Figure 3B). Raster plots show local bursts in individual neurons that did not propagate across the network starting from 28 dpi (Figure 3C and Supplementary Video 6). Synchronized network bursts were first detected around 49 dpi and developed into clear and synchronized burst features in later weeks around 63 dpi. These synchronized network bursts appeared with higher frequencies as the network developed further (Figure 3C and Supplementary Video 6).

The number of active neurons increased with culture age (from 272.3 ± 46.51 per HD-MEA on 22 dpi to 613.3 ± 83.32 on 49 dpi, and to its peak on 83 dpi with 870.0 ± 59.73 neurons, *p* < 0.05 vs. 22 dpi; Figure 3D). The number of neurons that contributed to network burst activity increased with culture age and reached 92.67 ± 21.34 neurons on 49 dpi and 213.3 ± 21.14 neurons on 90 dpi from only 6 neurons on 22 dpi (*p* < 0.05 and *p* < 0.001; Figure 3D). The average AP frequency was calculated by dividing the total number of APs to the recording period (Figure 3E). The AP firing rate reached its peak around 49 dpi (2.69 ± 0.07 Hz, *p* < 0.05 vs. 22 dpi with 1.55 ± 0.073 Hz, Figure 3E) and slightly declined in the following month (1.96 ± 0.05 Hz at 90 dpi, Figure 3E). The analysis of frequency domains for all recording days demonstrated that more than 51.69% of neurons tended to fire an average frequency below 2 Hz, while average firing frequencies of more than 5 Hz were detected only in less than 22.08% of neurons (Figure 3F).

Burst frequency exhibited a steady increase with culture age and reached from 0.01 ± 0.00 Hz on 22 dpi to 0.07 ± 0.01 Hz at 49 dpi and 0.23 ± 0.01 Hz on 90 dpi (*p* < 0.001 vs. 22 dpi, Figure 3G). Burst duration showed a sharp increase between 42 and 49 dpi (0.05 ± 0.01 s and 0.27 ± 0.02, respectively, *p* < 0.001; Figure 3H). Burst duration remained higher until 69 dpi (0.29 ± 0.01) and declined in following weeks (0.14 ± 0.01 on 90 dpi; Figure 3H) but were still significantly higher than on 22 dpi (*p* < 0.05). The percentage of APs that appear inside burst events increased with culture age and reached its peak around 69 dpi (55.51% of all APs, *p* < 0.001 vs. 22 dpi with 1.6 ± 0.39%, Figure 3I) and remained high for the rest of the study (Figure 3I).

### Development of instantaneous firing rate and fast spiking neurons

**FIGURE 4:**
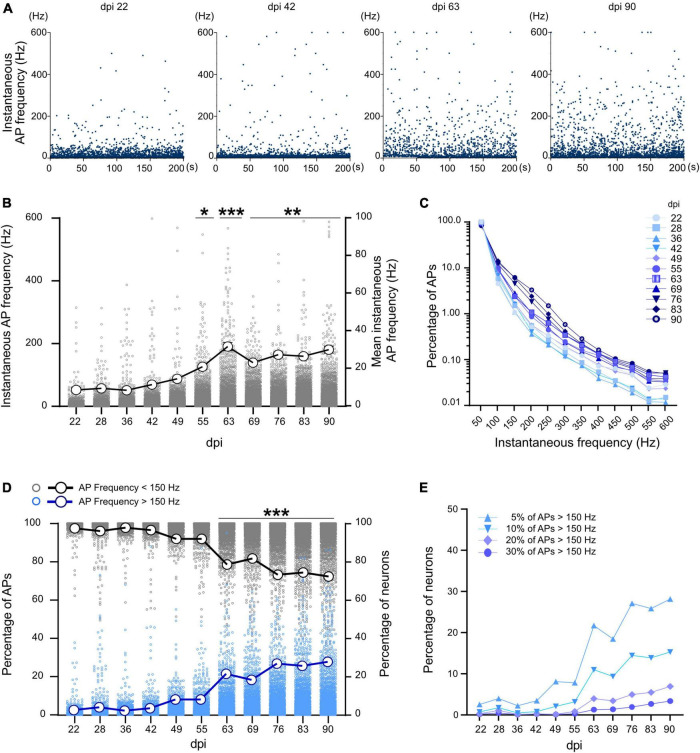
Instantaneous firing frequency in developing neuronal networks. **(A)** Instantaneous frequency of individual APs (spiking events) in a selected neuron at different time points. Each data point represents the instantaneous frequency of specific pair of APs during a 200 s recording period. **(B)** Average instantaneous frequency at different time points. Each data point represents the average instantaneous frequency of a neuron at particular dpi. Mean of the average frequencies in all neurons represented by large circles and thick line. **(C)** Distribution of the percentage of spiking events over the instantaneous frequency domains at different days (*n* > 400,000 events per dpi, *N* = 3 MEAs). **(D)** Percentage of APs with more than 150 Hz (blue) and less than 150 Hz (gray) in all neurons (*n* > 1,064 neurons). The thick blue line represents percentage of neurons with at least 5% of high frequency (>150 Hz) spiking events and the thick black line represents the percentage of the neurons with at least 95% low frequency spiking events (<150 Hz). **(E)** Percentage of neurons which have 5, 10, 20, or 30% of their spiking events in the high frequency domain (<150 Hz). Data between days were compared based on Kruskal-Wallis test followed by Dunn’s multiple comparison test. *p < 0.05, **p < 0.01, and ***p < 0.001 vs. 22, 28, 36, and 42 dpi.

As inhibitory neurons are fast-spiking and capable of firing in frequencies above 100 Hz, we extracted the spiking profile of neuronal activity using instantaneous firing rates. The average AP frequency during the whole recording period represents the mixture of active and silent states of neuronal activity (section “Long-term development of spontaneous activity and burst features in iNGN-derived neuronal networks”). The instantaneous firing frequency, on the other hand, is calculated based on individual ISIs between AP pairs, and provides detailed temporal information about neuronal activity. Instantaneous firing rate data enables to identify the maximal firing frequency of neurons. Average and maximum instantaneous frequency (*n* = 3 MEAs and more than 1,065 neurons) showed an increase during 3 months from 8.44 ± 0.62 Hz and 315 Hz at 22 dpi to 29.88 ± 0.93Hz and 588.1 Hz at 90 dpi, respectively (*p* < 0.001, Figures 4A,B). A sharp increase in average and maximum instantaneous frequency was observed after 63 dpi (31.57 ± 1.12Hz and 567.9 Hz, respectively, *p* < 0.001 vs. 22 dpi, Figure 4B). Percentage of high frequency spiking events (150–600 Hz) increased from 2.66% on 22 dpi to 11.33% on 90 dpi (Figure 4C). The percentage of neurons with at least 5% high frequency spiking events (>150 Hz) increased after 63 dpi (reached from 2.52% on 22 dpi to 27.74% on 90 dpi, *p* < 0.001, Figures 4D,E).

### Effect of AMPA and NMDA receptor antagonist on network function

**FIGURE 5:**
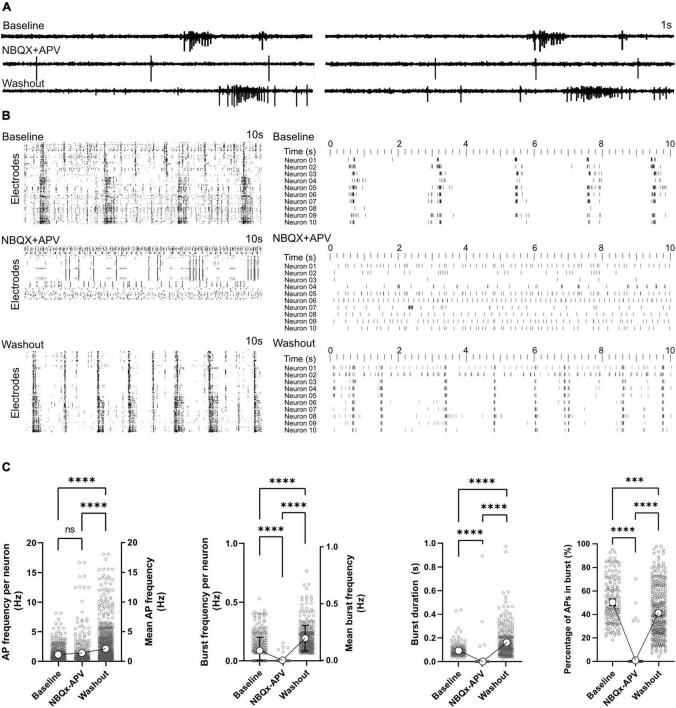
Effect of AMPA and NMDA receptor antagonists on network function. AMPA and NMDA receptors were blocked by NBQX+APV in two HD-MEAs on 118 dpi. Activity was recorded and analyzed for 5 min in the baseline, treatment and washout conditions. **(A)** Signal traces in two electrodes at baseline, under NBQX+APV treatment and washout. **(B)** Ten second raster plot of activity in all active electrodes (left) and 10 selected neurons (right) in the three different conditions with each row representing an electrode or neuron. **(C)** AP and burst frequency, burst duration and percentage of APs appearing in burst events were compared between the three conditions using Kruskal-Wallis test followed by Dunn’s multiple comparison test. ^***^p < 0.001 and ^*⁣*⁣**^p < 0.0001. Each point represents the corresponding activity feature in one neuron.

Activity scans and network analyses were performed before, during and after the treatment with glutamatergic synapse blockers (NBQX+APV). Signal traces in electrodes showed that in the presence of the NBQX+APV, synchronized burst features vanished and only individual APs and localized bursts (Figure 5A) were detectable. Raster plots of activity also indicated the lack of synchronized burst activity in NBQX+APV treated conditions (Figure 5B). Burst features re-appeared in the recorded signal profile and raster plots after washing the NBQX+APV out (Figures 5A,B). The number of active neurons decreased from 595 neurons at baseline to 407 neurons in presence of the NBQX+APV (Supplementary Figure 5). AP firing frequency of remaining active neurons was not affected by NBQX+APV treatment (0.13 ± 0.04Hz in baseline and 1.39 ± 0.11Hz in treated condition, *p* = 0.35; Figure 5C). After washout, AP frequency increased to 2.12 ± 0.09 Hz (*p* < 0.0001 vs. baseline and treated condition). Only few neurons out of thousands of neurons showed burst activity in the presence of NBQX+APV (Figures 5B,C). Burst frequency, duration and percentage of APs in the burst decreased significantly by NBQX+APV treatment (*p* < 0.0001 vs. baseline, Figure 5C). Burst parameters increased after washout (burst frequency and duration: *p* < 0.0001 vs. baseline and percentage of APs in burst: *p* < 0.001; Figure 5C). It has been shown that media exchange affects the network activity which lasts for approximately 1 day. Unfortunately, in the current experiment design the activity changes related to the washout were inevitable. Whether the activity changes after chemical treatment are a result of releasing inhibitors from synapses or indirectly from the media exchange is hard to explain. However, during the chemical treatment there was no media exchange and effects are directly related to the applied blockers.

### Effect of GABA-A receptor antagonist on network function

**FIGURE 6:**
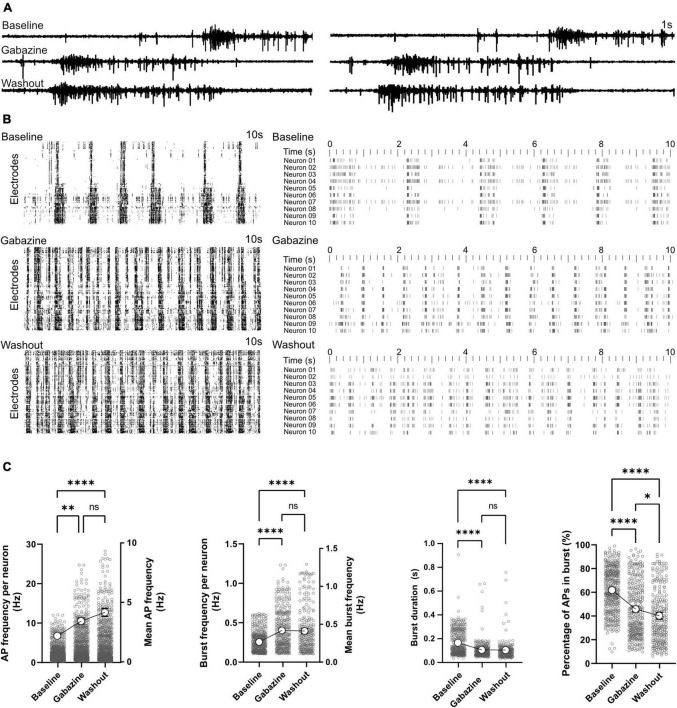
Effect of GABA-A receptor antagonist on network function. GABA-A receptors were blocked by gabazine in two HD-MEAs on 120 dpi. Baseline activity, activity under gabazine treatment and activity after washout were recorded. **(A)** Signal traces recorded from two electrodes at baseline, under gabazine and after washout. **(B)** Raster plot of activity in individual electrodes (left) and 10 selected neurons (right) in the three different conditions with each row representing an electrode or neuron. **(C)** AP and burst frequency, burst duration and percentage of APs appearing in burst events were compared between the three conditions using Kruskal-Wallis test followed by Dunn’s multiple comparison test. *p < 0.05, ^**^p < 0.01, ^*⁣*⁣**^p < 0.0001, and ns, non-significant. Each point represents the corresponding activity feature in one neuron.

Activity scans and network analysis were performed at baseline, during and after treatment with GABAergic synapse antagonist gabazine. Representative signal traces of two electrodes in the presence of gabazine are shown in Figure 6A. Activity raster plots revealed an increase in network burst rate when treated with gabazine (Figure 6B). Based on the raster plot profile, this increase in bursting rate remains stable after washout (Figure 6B). Statistical analysis of our time stamp data showed a significant increase in neuronal AP firing frequencies in the presence of gabazine (2.18 ± 0.07 Hz in baseline and 3.43 ± 0.12 Hz in treated condition, *p* < 0.01; Figure 6C). Neuronal AP frequencies remained higher after washout and reached 4.16 ± 0.17 Hz (*p* < 0.0001 vs. baseline and *p* = 0.36 vs. gabazine treated condition). The burst frequency was elevated by gabazine treatment (0.42 ± 0.01 Hz vs. 0.27 ± 0.01 Hz in baseline, *p* < 0.0001; Figure 6C) and remained higher after washout (0.41 ± 0.02Hz, *p* < 0.0001 vs. baseline). The duration of bursts decreased by gabazine treatment from 0.16 ± 0.01s to 0.10 ± 0.01 s (*p* < 0.0001; Figure 6C). The percentage of APs in bursts also decreased by gabazine treatment (45.25 ± 1.23% vs. 61.33 ± 0.99% in baseline, *p* < 0.0001; Figure 6C). After washout, burst duration and percentage of APs in bursts remained lower than baseline conditions (*p* < 0.0001; Figure 6C).

## Discussion

A key step to enable hiPSC-derived neuronal cultures as reliable *in vitro* models of developing brain circuits is to generate functionally robust networks (McCready et al., 2022). This needs to be accompanied by enhanced access to the network activity and morphology data with optimal spatial and temporal resolution at the neuronal cell level (Dragas et al., 2017; Diggelmann et al., 2018; Yuan et al., 2020; Ronchi et al., 2021). Most importantly, these networks require long-term viability to allow proper maturation of the network structure and function, and to measure their developmental features for an extended period (Saalfrank et al., 2015). Previous studies on hiPSC-derived networks were either limited to short-term evaluation of less than a month (Ronchi et al., 2021; Akarca et al., 2022) or probing the activity with conventional MEAs with limited number of electrodes (Odawara et al., 2014, 2016; Hyvärinen et al., 2019; Lu et al., 2019; Schmieder et al., 2022). Here we exploited the advantages of HD-MEAs and our established long-term culturing protocol for hiPSC-derived networks (Klapper et al., 2017; Sauter et al., 2019; Schmieder et al., 2022) to extract functional and morphological data of developing hiPSC-derived networks for 3 months. We constantly applied astrocyte-conditioned medium to the neuronal cultures to improve their functional maturation (Klapper et al., 2017; Taga et al., 2019).

To the best of our knowledge, long-term HD-MEA-based recordings from hiPSC-derived networks have only been studied by Amin et al. (2016), where they reported changes in AP frequency but mainly focused on network responses to electrical stimulation. In our previous work, we generated long-term hiPSC-derived networks on standard MEAs with 60 electrodes (Schmieder et al., 2022). Time-lapse images from these cultures at different weeks revealed large-scale changes in the network morphology (Supplementary Video 4). These inherent changes in developing networks with continuous movement of neuronal cells on low-density MEA substrates can affect the quality of recorded data such that functional neurons are no longer captured since they moved too far from the electrodes to be recorded. Here we tried to overcome this issue by using HD-MEAs, which enable capturing active regions on the whole sensor area with higher spatial resolution. Our data demonstrated that HD-MEAs are capable of tracking dynamic changes in neuronal position and network morphology (Figure 2 and Supplementary Videos 1–3). Therefore, to obtain reliable functional data from long-term networks, a full activity scan prior to the network recording is crucial to get a complete network activity map and identify the position of the neurons and clusters. This enables to record from identical neurons over months and to robustly track their functional phenotype at different culture ages. Nevertheless, limitations of the technology and used scanning assays is that only 1,024 electrodes can be recorded simultaneously thus making it possible to only capture a part of the network. Also, scanning the whole electrode area requires long recording sessions.

Physical expansion of the brain volume during development requires an optimized wiring economy to facilitate communication between neurons in close vicinity or large distance (Wang and Clandinin, 2016; Casanova and Casanova, 2019). This wiring principle leads to modular and clustered network structures in which neurons and regions performing functionally related tasks are typically adjacent to decrease the wiring costs (Bassett and Bullmore, 2006; Wang and Clandinin, 2016; Casanova and Casanova, 2019). Examples of structural clustering are cortical folding and gyrification in order to bring structurally separated regions together (Wang and Clandinin, 2016; Chavoshnejad et al., 2021). Separation of gray and white matter in vertebrates is another example of how local circuits or modules are clustered but connected through long-range axons (Wang and Clandinin, 2016; Richards and Van Hooser, 2018; Martinez and Sprecher, 2020). In the present work, morphological images as well as functional activity maps revealed that individual neurons are pulled together to form small clusters beginning in the second month of culturing. Larger clusters were formed from smaller clusters merging together beginning in the third month (Figure 2 and Supplementary Videos 1, 2). These clusters were connected through axonal bundles, mimicking the primitive regionalization and folding of the developing human brain (Figure 2). In a previous study, Tibau et al. (2020) generated clustered and semi-clustered rat cortical networks and compared their functional features with homogenously distributed cortical networks for 2 weeks. Aggregated circuits showed higher burst rates and functional connectivity features, which emphasizes the influence of neuronal spatial arrangement on network function (Tibau et al., 2020). Modular networks engineered in microfluidic devices with clustered network architecture and controlled connectivity between clusters have also shown an enhanced activity profile (Nam et al., 2004; Levy et al., 2012; Marconi et al., 2012; Yamamoto et al., 2018; Park et al., 2021). These results are in accordance with our long-term data that showed enhanced activity and burst parameters correlating with morphological changes from homogenously distributed to clustered networks. Thus, it can be hypothesized that the observed clustering behavior is a result of the inherent developmental tendency of neurons to form functional clusters in their vicinity and to bundle the axons for long-range communication. Even though the chip surface is prepared to allow good adherence of the cells, migration is still occurring further supporting this hypothesis. Also, clustering and bundle formation is observed in other neuronal *in vitro* cultures as well (Segev et al., 2003; Tibau et al., 2020; Antonello et al., 2022).

We continuously extracted electrophysiology data of long-term cultures. iNGN cells have been shown to become of neuronal type with a very high efficiency upon doxycycline induction as confirmed by FoxG1 and Map2- staining and RT-qPCR analysis (Lu et al., 2019). We monitored healthy development of the cultures by microscopy. Spike sorting was applied to extract the activity profiles of individual neurons in each recording. These data demonstrated that activity in developing iNGN-EGFP-derived networks evolved from single and sparse APs in the first month post induction to localized burst activity in the second month and developed to synchronized bursts across the network in the third month. Burst features followed separate trajectories during the development. While burst frequency showed a steady increase, burst duration experienced a peak in the second month and slightly declined in the third month. The percentage of APs that occur in burst events remained high after reaching a peak around 69 dpi. Previous studies on developing prenatal and postnatal human and rodent brains, and *in vitro* primary networks indicated spontaneous synchronized network burst activity as a key functional feature of developing neocortical circuits (Khazipov and Luhmann, 2006; Kilb et al., 2011; Luhmann et al., 2016). Correlated burst activity facilitates functional maturation of the network and is essential for the formation of cortical circuits (Khazipov and Luhmann, 2006). Bursts with a high content of APs reliably cross through weak synapses and trigger postsynaptic activity that is necessary for enhancing neuron-to-neuron synaptic communication (Zeldenrust et al., 2018). Here, we tested the role of excitatory synapses on the presence of burst features by inhibition of the glutamatergic synapses. This erased the synchronized burst features from our recordings and decreased number of the active neurons. Sustained AP firing and a constant frequency in presence of NBQX+APV compared to the baseline suggested that neurons are still active but are effectively blocked from synaptic communication. It can be concluded that excitatory synapses are the main feature through which network bursts are mediated in iNGN cultures.

In contrast to the earlier development of glutamatergic transmission in embryonic stages within rodent and human brains, the GABAergic system requires longer time for maturation (Bagasrawala et al., 2017; Warm et al., 2022). Delayed maturation of GABAergic synapses in the postnatal period overlaps with the emergence of strong burst activity in the developing mammalian brain (Khazipov and Luhmann, 2006; Luhmann et al., 2016; Luhmann and Khazipov, 2018). Lu et al. (2019), have shown that the percentage of GABAergic neurons in developing iNGN-derived networks increases by culture age and reaches 2.3% at 30 dpi. They also showed that GABA receptor antagonist Picrotoxin (PTX) increases the firing rate and burst frequency. Early during brain development, GABAergic synaptic transmission is excitatory and switches to mature inhibitory neurotransmission in the early postnatal period (Ganguly et al., 2001). The GABAergic switch has also been demonstrated in hiPSCs-derived neuronal organoids around day 40 (Zafeiriou et al., 2020). In our study, iNGN-EGFP networks at 120 dpi were used for studying GABA signaling that gave sufficient time for maturation of these synapses. Our results showed that inhibition of the GABA-A receptors induced an increase in AP and burst frequency. These results demonstrated that at 120 dpi the GABAergic system has already switched into inhibitory neurotransmission.

A major part of the cortical inhibitory neurons are fast spiking GABAergic neurons that are functionally critical for modulating excitatory activity (Galarreta and Hestrin, 2002). Normally, these inhibitory neurons are identified by their fast responses to current injection (Damodaran et al., 2014). Maximal firing rates of fast spiking neurons have been determined based on their instantaneous frequency in brain slices of human, monkey, and mouse and as well in behaving monkey and mouse (Wang et al., 2016). In the current work we showed that in a small fraction of neuronal cells instantaneous AP frequency was more than 150 Hz. Percentage of fast spiking events and neurons increased sharply around 63 dpi that coincides with maturation of the burst features. These data together indicated that the emergence of the GABAergic system and its maturation regulates the characteristics of network bursts. This is in line with results obtained from the developing postnatal brain that demonstrate involvement of GABAergic signaling on modulating the spontaneous and sensory-evoked burst activity of neuronal circuits (Bonifazi et al., 2009; Butt et al., 2017; Warm et al., 2022). Other *in vitro* studies in which different ratios of inhibitory and excitatory neurons in the culture caused changes in bursting and overall network behavior support our findings (Chen and Dzakpasu, 2010). Especially the interplay of excitatory NMDA and inhibitory GABA receptors are essential for the formation of different patterns of synchronized network activity (Teppola et al., 2019). This is in line with our data that suggested an effect of GABAergic system on synchronized network bursts development. Due to the strong influence of GABA antagonists on network activity it can be hypothesized that also network composition and cell identity are changing over time. Moreover, it still must be dissected how the clustering of cells and cell maturation are influencing the network activity or if and how they are connected.

## Conclusion

Our data indicated the importance of long-term continuous data readouts from developing hiPSC-derived networks to obtain robust results and show functional dynamics of developing networks in detail. Long-term tracking of network morphology revealed constant changes in the position of neuronal cells and clusters that were reflected in network activity images obtained by HD-MEA recordings. Neuronal migration, formation of the clusters and extended axonal bundles for 3 months resemble morphological changes in expanding and folding cortical networks. Such changes in morphology and network coordinates must be taken into account when designing *in vitro* experiments. This is especially the case when looking at network functional connectivity maps since changes in network morphology directly influence functional connectivity and cannot be assumed as stable parameter over time. Our data exhibited in detail the emergence and maturation of the spontaneous and synchronized burst activity resembling the functional features of the developing brain during the prenatal and postnatal periods. Large variations in burst features during *in vitro* network development suggested the criticality of selecting limited time points in short-term studies to predict network functional phenotype. This can become even more critical when functional phenotype of healthy or diseased networks are predicted based on snapshots of short-term data without considering long-term network maturation profiles in the analysis. Further analysis is needed to see how closely *in vitro* network development is following the *in vivo* states with morphology and potentially cell identity changing simultaneously. It is also noteworthy that iNGN networks functionally mature within 3 months, displaying robust spontaneous activity. As these networks morphologically drift over time, HD-MEA have the resolution to detect these movements as well as have the resolution to facilitate continuous recordings. Therefore, using HD-MEA significantly increases the functional readouts of human stem cell-derived neuronal networks, i.e., one needs less MEA cultures per cell line and experiment.

## Data availability statement

The original contributions presented in the study are included in the article/Supplementary material, further inquiries can be directed to the corresponding author.

## Author contributions

VB: conceptualization. VB, JC, RH, and JS: methodology. JS, FS, and RH: software and validation. RH and JS: formal analysis, investigation, visualization, and data curation. RH, JS, and VB: writing-original draft. RH, JS, FS, JC, and VB: writing—review and editing. VB and JC: supervision, project administration, resources, and funding acquisition. All authors contributed to the article and approved the submitted version.

## References

[B1] Akarca D. Dunn A. W. E. Hornauer P. J. Ronchi S. Fiscella M. Wang C. (2022). Homophilic wiring principles underpin neuronal network topology in vitro. *bioRxiv* [Preprint]. 10.1101/2022.03.09.483605 PMC12563572 40626695

[B2] Amin H. Maccione A. Marinaro F. Zordan S. Nieus T. Berdondini L. (2016). Electrical responses and spontaneous activity of human iPS-derived neuronal networks characterized for 3-month culture with 4096-electrode arrays. *Front. Neurosci.* 10:121. 10.3389/fnins.2016.00121 27065786 PMC4811967

[B3] Antonello P. C. Varley T. F. Beggs J. Porcionatto M. Sporns O. Faber J. (2022). Self-organization of in vitro neuronal assemblies drives to complex network topology. *Elife* 11:e74921. 10.7554/elife.74921 35708741 PMC9203058

[B4] Ardhanareeswaran K. Mariani J. Coppola G. Abyzov A. Vaccarino F. M. (2017). Human induced pluripotent stem cells for modelling neurodevelopmental disorders. *Nat. Rev. Neurol.* 13 265–278. 10.1038/nrneurol.2017.45 28418023 PMC5782822

[B5] Bagasrawala I. Memi F. Radonjić N. V. Zecevic N. (2017). N -Methyl d -Aspartate receptor expression patterns in the human fetal cerebral cortex. *Cereb. Cortex* 27 5041–5053. 10.1093/cercor/bhw289 27664962 PMC6077866

[B6] Ballini M. Muller J. Livi P. Chen Y. Frey U. Stettler A. (2014). A 1024-channel CMOS microelectrode array with 26,400 electrodes for recording and stimulation of electrogenic cells in vitro. *IEEE J. Solid State Circuits* 49 2705–2719. 10.1109/JSSC.2014.2359219 28502989 PMC5424881

[B7] Bassett D. S. Bullmore E. (2006). Small-world brain networks. *Neuroscientist* 12 512–523. 10.1177/1073858406293182 17079517

[B8] Bassett D. S. Gazzaniga M. S. (2011). Understanding complexity in the human brain. *Trends Cogn. Sci.* 15 200–209. 10.1016/j.tics.2011.03.006 21497128 PMC3170818

[B9] Bassett D. S. Sporns O. (2017). Network neuroscience. *Nat. Neurosci.* 20 353–364. 10.1038/nn.4502 28230844 PMC5485642

[B10] Berdondini L. Imfeld K. MacCione A. Tedesco M. Neukom S. Koudelka-Hep M. (2009). Active pixel sensor array for high spatio-temporal resolution electrophysiological recordings from single cell to large scale neuronal networks. *Lab Chip* 9 2644–2651. 10.1039/b907394a 19704979

[B11] Bonifazi P. Goldin M. Picardo M. A. Jorquera I. Cattani A. Bianconi G. (2009). GABAergic hub neurons orchestrate synchrony in developing hippocampal networks. *Science* 326 1419–1424. 10.1126/science.1175509 19965761

[B12] Buccino A. P. Hurwitz C. L. Garcia S. Magland J. Siegle J. H. Hurwitz R. (2020). Spikeinterface, a unified framework for spike sorting. *Elife* 9 1–24. 10.7554/eLife.61834 33170122 PMC7704107

[B13] Budday S. Steinmann P. Kuhl E. (2015). Physical biology of human brain development. *Front. Cell Neurosci.* 9:257. 10.3389/fncel.2015.00257 26217183 PMC4495345

[B14] Busskamp V. Lewis N. E. Guye P. Ng A. H. Shipman S. L. Byrne S. M. (2014). Rapid neurogenesis through transcriptional activation in human stem cells. *Mol. Syst. Biol.* 10:760. 10.15252/msb.20145508 25403753 PMC4299601

[B15] Butt S. J. Stacey J. A. Teramoto Y. Vagnoni C. (2017). A role for GABAergic interneuron diversity in circuit development and plasticity of the neonatal cerebral cortex. *Curr. Opin. Neurobiol.* 43 149–155. 10.1016/j.conb.2017.03.011 28399421

[B16] Canals I. Ginisty A. Quist E. Timmerman R. Fritze J. Miskinyte G. (2018). Rapid and efficient induction of functional astrocytes from human pluripotent stem cells. *Nat. Methods* 15 693–696. 10.1038/s41592-018-0103-2 30127505

[B17] Casanova M. F. Casanova E. L. (2019). The modular organization of the cerebral cortex: Evolutionary significance and possible links to neurodevelopmental conditions. *J. Comp. Neurol.* 527 1720–1730. 10.1002/cne.24554 30303529 PMC6784310

[B18] Chavoshnejad P. Li X. Zhang S. Dai W. Vasung L. Liu T. (2021). Role of axonal fibers in the cortical folding patterns: A tale of variability and regularity. *Brain Multiphys.* 2:100029. 10.1016/j.brain.2021.100029

[B19] Chen X. Dzakpasu R. (2010). Observed network dynamics from altering the balance between excitatory and inhibitory neurons in cultured networks. *Phys. Rev. E* 82:31907. 10.1103/PhysRevE.82.031907 21230108

[B20] Corlew R. Bosma M. M. Moody W. J. (2004). Spontaneous, synchronous electrical activity in neonatal mouse cortical neurones. *J. Physiol.* 560 377–390. 10.1113/jphysiol.2004.071621 15297578 PMC1665264

[B21] Cotterill E. Eglen S. J. (2019). “Burst detection methods,” in *Advances in neurobiology*, eds Chiappalone M. Pasquale V. Frega M. (Cham: Springer International Publishing), 185–206. 10.1007/978-3-030-11135-9_8 31073937

[B22] Craig A. M. Graf E. R. Linhoff M. W. (2006). How to build a central synapse: Clues from cell culture. *Trends Neurosci.* 29 8–20. 10.1016/j.tins.2005.11.002 16337695 PMC2820512

[B23] Craik F. I. M. Bialystok E. (2006). Cognition through the lifespan: Mechanisms of change. *Trends Cogn. Sci.* 10 131–138. 10.1016/j.tics.2006.01.007 16460992

[B24] Damodaran S. Evans R. C. Blackwell K. T. (2014). Synchronized firing of fast-spiking interneurons is critical to maintain balanced firing between direct and indirect pathway neurons of the striatum. *J. Neurophysiol.* 111 836–848. 10.1152/jn.00382.2013 24304860 PMC3921391

[B25] Diggelmann R. Fiscella M. Hierlemann A. Franke F. (2018). Automatic spike sorting for high-density microelectrode arrays. *J. Neurophysiol.* 120 3155–3171. 10.1152/jn.00803.2017 30207864 PMC6314465

[B26] Dragas J. Viswam V. Shadmani A. Chen Y. Bounik R. Stettler A. (2017). In vitro multi-functional microelectrode array featuring 59 760 electrodes, 2048 electrophysiology channels, stimulation, impedance measurement, and neurotransmitter detection channels. *IEEE J. Solid State Circuits* 52 1576–1590. 10.1109/JSSC.2017.2686580 28579632 PMC5447818

[B27] Galarreta M. Hestrin S. (2002). Electrical and chemical synapses among parvalbumin fast-spiking GABAergic interneurons in adult mouse neocortex. *Proc. Natl. Acad. Sci. U.S.A.* 99 12438–12443. 10.1073/pnas.192159599 12213962 PMC129463

[B28] Ganguly K. Schinder A. F. Wong S. T. Poo M. (2001). GABA itself promotes the developmental switch of neuronal GABAergic responses from excitation to inhibition. *Cell* 105 521–532. 10.1016/S0092-8674(01)00341-5 11371348

[B29] Gozdas E. Holland S. K. Altaye M. (2019). Developmental changes in functional brain networks from birth through adolescence. *Hum. Brain Mapp.* 40 1434–1444. 10.1002/hbm.24457 30582266 PMC6865712

[B30] Habibey R. Golabchi A. Blau A. (2015a). “Microchannel scaffolds for neural signal acquisition and analysis,” in *Neurotechnology, electronics, and informatics*, eds Londral A. R. Encarnação P. Rovira J. L. P. (Cham: Springer International Publishing), 47–64.

[B31] Habibey R. Golabchi A. Latifi S. Difato F. Blau A. (2015b). A microchannel device tailored to laser axotomy and long-term microelectrode array electrophysiology of functional regeneration. *Lab Chip* 15 4578–4590. 10.1039/c5lc01027f 26507288

[B32] Habibey R. Latifi S. Mousavi H. Pesce M. Arab-Tehrany E. Blau A. (2017). A multielectrode array microchannel platform reveals both transient and slow changes in axonal conduction velocity. *Sci. Rep.* 7:8558. 10.1038/s41598-017-09033-3 28819130 PMC5561146

[B33] Habibey R. Sharma K. Swiersy A. Busskamp V. (2020). Optogenetics for neural transplant manipulation and functional analysis. *Biochem. Biophys. Res. Commun.* 527 343–349. 10.1016/j.bbrc.2020.01.141 32033753

[B34] Habibey R. Striebel J. Sharma K. Busskamp V. (2022). Optogenetic control of human stem cell-derived neurons. *Methods Mol. Biol.* 2501 339–360. 10.1007/978-1-0716-2329-9_17 35857237

[B35] Hattori N. (2014). Cerebral organoids model human brain development and microcephaly. *Mov. Disord.* 29 185–185. 10.1002/mds.25740 24375826

[B36] Hockemeyer D. Jaenisch R. (2016). Induced pluripotent stem cells meet genome editing. *Cell Stem Cell* 18 573–586. 10.1016/j.stem.2016.04.013 27152442 PMC4871596

[B37] Huber L. Finn E. S. Chai Y. Goebel R. Stirnberg R. Stöcker T. (2021). Layer-dependent functional connectivity methods. *Prog. Neurobiol.* 207:101835. 10.1016/j.pneurobio.2020.101835 32512115 PMC11800141

[B38] Hyvärinen T. Hyysalo A. Kapucu F. E. Aarnos L. Vinogradov A. Eglen S. J. (2019). Functional characterization of human pluripotent stem cell-derived cortical networks differentiated on laminin-521 substrate: Comparison to rat cortical cultures. *Sci. Rep.* 9:17125. 10.1038/s41598-019-53647-8 31748598 PMC6868015

[B39] Ilic D. Ogilvie C. (2017). Concise review: Human embryonic stem cells—What have we done? What are we doing? Where are we going? *Stem Cells* 35 17–25. 10.1002/stem.2450 27350255

[B40] Jiang X. Nardelli J. (2016). Cellular and molecular introduction to brain development. *Neurobiol. Dis.* 92 3–17. 10.1016/j.nbd.2015.07.007 26184894 PMC4720585

[B41] Khazipov R. Luhmann H. J. (2006). Early patterns of electrical activity in the developing cerebral cortex of humans and rodents. *Trends Neurosci.* 29 414–418. 10.1016/j.tins.2006.05.007 16713634

[B42] Kilb W. Kirischuk S. Luhmann H. J. (2011). Electrical activity patterns and the functional maturation of the neocortex. *Eur. J. Neurosci.* 34 1677–1686. 10.1111/j.1460-9568.2011.07878.x 22103424

[B43] Kim J. Y. Paredes M. F. (2021). Implications of extended inhibitory neuron development. *Int. J. Mol. Sci.* 22:5113. 10.3390/ijms22105113 34066025 PMC8150951

[B44] Kirwan P. Turner-Bridger B. Peter M. Momoh A. Arambepola D. Robinson H. P. C. (2015). Development and function of human cerebral cortex neural networks from pluripotent stem cells in vitro. *Development* 142 3178–3187. 10.1242/dev.123851 26395144 PMC4582178

[B45] Klapper S. D. Sauter E. J. Swiersy A. Hyman M. A. E. Bamann C. Bamberg E. (2017). On-demand optogenetic activation of human stem-cell-derived neurons. *Sci. Rep.* 7:14450. 10.1038/s41598-017-14827-6 29089561 PMC5663899

[B46] Kriegeskorte N. Douglas P. K. (2018). Cognitive computational neuroscience. *Nat. Neurosci.* 21 1148–1160. 10.1038/s41593-018-0210-5 30127428 PMC6706072

[B47] Kutsche L. K. Gysi D. M. Fallmann J. Lenk K. Petri R. Swiersy A. (2018). Combined experimental and system-level analyses reveal the complex regulatory network of miR-124 during human neurogenesis. *Cell Syst.* 7 438–452.e8. 10.1016/j.cels.2018.08.011 30292704 PMC6205824

[B48] Lam R. S. Töpfer F. M. Wood P. G. Busskamp V. Bamberg E. (2017). Functional maturation of human stem cell-derived neurons in long-term cultures. *PLoS One* 12:e0169506. 10.1371/journal.pone.0169506 28052116 PMC5215418

[B49] Latifi S. Mitchell S. Habibey R. Hosseini F. Donzis E. Estrada-Sanchez A. M. (2020). Neuronal network topology indicates distinct recovery processes after stroke. *Cereb. Cortex* 30 6363–6375. 10.1093/cercor/bhaa191 32728724 PMC8257065

[B50] Latifi S. Tamayol A. Habibey R. Sabzevari R. Kahn C. Geny D. (2016). Natural lecithin promotes neural network complexity and activity. *Sci. Rep.* 6:25777. 10.1038/srep25777 27228907 PMC4882550

[B51] Legendy C. R. Salcman M. (1985). Bursts and recurrences of bursts in the spike trains of spontaneously active striate cortex neurons. *J. Neurophysiol.* 53 926–939. 10.1152/jn.1985.53.4.926 3998798

[B52] Levakov G. Faskowitz J. Avidan G. Sporns O. (2021). Mapping individual differences across brain network structure to function and behavior with connectome embedding. *Neuroimage* 242:118469. 10.1016/j.neuroimage.2021.118469 34390875 PMC8464439

[B53] Levy O. Ziv N. E. Marom S. (2012). Enhancement of neural representation capacity by modular architecture in networks of cortical neurons. *Eur. J. Neurosci.* 35 1753–1760. 10.1111/j.1460-9568.2012.08094.x 22507055

[B54] Li J. Curley W. H. Guerin B. Dougherty D. D. Dalca A. V. Fischl B. (2021). Mapping the subcortical connectivity of the human default mode network. *Neuroimage* 245:118758. 10.1016/j.neuroimage.2021.118758 34838949 PMC8945548

[B55] Lu C. Shi X. Allen A. Baez-Nieto D. Nikish A. Sanjana N. E. (2019). Overexpression of NEUROG2 and NEUROG1 in human embryonic stem cells produces a network of excitatory and inhibitory neurons. *FASEB J.* 33 5287–5299. 10.1096/fj.201801110RR 30698461 PMC6436650

[B56] Luhmann H. J. Khazipov R. (2018). Neuronal activity patterns in the developing barrel cortex. *Neuroscience* 368 256–267. 10.1016/j.neuroscience.2017.05.025 28528963

[B57] Luhmann H. J. Sinning A. Yang J. W. Reyes-Puerta V. Stüttgen M. C. Kirischuk S. (2016). Spontaneous neuronal activity in developing neocortical networks: From single cells to large-scale interactions. *Front. Neural Circuits* 10:40. 10.3389/fncir.2016.00040 27252626 PMC4877528

[B58] Mannal N. Kleiner K. Fauler M. Dougalis A. Poetschke C. Liss B. (2021). Multi-electrode array analysis identifies complex dopamine responses and glucose sensing properties of substantia nigra neurons in mouse brain slices. *Front. Synaptic Neurosci.* 13:635050. 10.3389/fnsyn.2021.635050 33716704 PMC7952765

[B59] Manzini A. Jones E. J. H. Charman T. Elsabbagh M. Johnson M. H. Singh I. (2021). Ethical dimensions of translational developmental neuroscience research in autism. *J. Child Psychol. Psychiatry Allied Discip.* 62 1363–1373. 10.1111/jcpp.13494 34405894 PMC7611913

[B60] Marconi E. Nieus T. Maccione A. Valente P. Simi A. Messa M. (2012). Emergent functional properties of neuronal networks with controlled topology. *PLoS One* 7:e34648. 10.1371/journal.pone.0034648 22493706 PMC3321036

[B61] Martinez P. Sprecher S. G. (2020). Of circuits and brains: The origin and diversification of neural architectures. *Front. Ecol. Evol.* 8:82. 10.3389/fevo.2020.00082

[B62] Matsuda N. Odawara A. Katoh H. Okuyama N. Yokoi R. Suzuki I. (2018). Detection of synchronized burst firing in cultured human induced pluripotent stem cell-derived neurons using a 4-step method. *Biochem. Biophys. Res. Commun.* 497 612–618. 10.1016/j.bbrc.2018.02.117 29454965

[B63] McCready F. P. Gordillo-Sampedro S. Pradeepan K. Martinez-Trujillo J. Ellis J. (2022). Multielectrode arrays for functional phenotyping of neurons from induced pluripotent stem cell models of neurodevelopmental disorders. *Biology (Basel)* 11:316. 10.3390/biology11020316 35205182 PMC8868577

[B64] Mezinska S. Gallagher L. Verbrugge M. Bunnik E. M. (2021). Ethical issues in genomics research on neurodevelopmental disorders: A critical interpretive review. *Hum. Genomics* 15:16. 10.1186/s40246-021-00317-4 33712057 PMC7953558

[B65] Mossink B. Verboven A. H. A. van Hugte E. J. H. Klein Gunnewiek T. M. Parodi G. Linda K. (2021). Human neuronal networks on micro-electrode arrays are a highly robust tool to study disease-specific genotype-phenotype correlations in vitro. *Stem Cell Rep.* 16 2182–2196. 10.1016/j.stemcr.2021.07.001 34329594 PMC8452490

[B66] Müller J. Ballini M. Livi P. Chen Y. Radivojevic M. Shadmani A. (2015). High-resolution CMOS MEA platform to study neurons at subcellular, cellular, and network levels. *Lab Chip* 15 2767–2780. 10.1039/c5lc00133a 25973786 PMC5421573

[B67] Nam Y. Chang J. Khatami D. Brewer G. J. Wheeler B. C. (2004). Patterning to enhance activity of cultured neuronal networks. *IEE Proc. Nanobiotechnol.* 151 109–115. 10.1049/ip-nbt:20040706 16475852

[B68] Ndyabawe K. Kisaalita W. S. (2019). Engineering microsystems to recapitulate brain physiology on a chip. *Drug Discov. Today* 24 1725–1730. 10.1016/j.drudis.2019.06.008 31226433

[B69] Ng A. H. M. Khoshakhlagh P. Rojo Arias J. E. Pasquini G. Wang K. Swiersy A. (2021). A comprehensive library of human transcription factors for cell fate engineering. *Nat. Biotechnol.* 39 510–519. 10.1038/s41587-020-0742-6 33257861 PMC7610615

[B70] Nikolakopoulou P. Rauti R. Voulgaris D. Shlomy I. Maoz B. M. Herland A. (2021). Recent progress in translational engineered in vitro models of the central nervous system. *Brain* 143 3181–3213. 10.1093/BRAIN/AWAA268 33020798 PMC7719033

[B71] Odawara A. Katoh H. Matsuda N. Suzuki I. (2016). Physiological maturation and drug responses of human induced pluripotent stem cell-derived cortical neuronal networks in long-term culture. *Sci. Rep.* 6:26181. 10.1038/srep26181 27188845 PMC4870631

[B72] Odawara A. Saitoh Y. Alhebshi A. H. Gotoh M. Suzuki I. (2014). Long-term electrophysiological activity and pharmacological response of a human induced pluripotent stem cell-derived neuron and astrocyte co-culture. *Biochem. Biophys. Res. Commun.* 443 1176–1181. 10.1016/j.bbrc.2013.12.142 24406164

[B73] Pachitariu M. Steinmetz N. Kadir S. Carandini M. Harris K. D. (2016). Kilosort: Realtime spike-sorting for extracellular electrophysiology with hundreds of channels. *bioRxiv* [Preprint]. 10.1101/061481

[B74] Pang Z. P. Yang N. Vierbuchen T. Ostermeier A. Fuentes D. R. Yang T. Q. (2011). Induction of human neuronal cells by defined transcription factors. *Nature* 476 220–223. 10.1038/nature10202 21617644 PMC3159048

[B75] Park M. U. Bae Y. Lee K. S. Song J. H. Lee S. M. Yoo K. H. (2021). Collective dynamics of neuronal activities in various modular networks. *Lab Chip* 21 951–961. 10.1039/d0lc01106a 33475100

[B76] Paşca S. P. (2018). The rise of three-dimensional human brain cultures. *Nature* 553 437–445. 10.1038/nature25032 29364288

[B77] Pastrana E. (2013). Focus on mapping the brain. *Nat. Methods* 10 481–481. 10.1038/nmeth.2509 23866324

[B78] Preibisch S. Saalfeld S. Tomancak P. (2009). Globally optimal stitching of tiled 3D microscopic image acquisitions. *Bioinformatics* 25 1463–1465. 10.1093/bioinformatics/btp184 19346324 PMC2682522

[B79] Raybaud C. Ahmad T. Rastegar N. Shroff M. Al Nassar M. (2013). The premature brain: Developmental and lesional anatomy. *Neuroradiology* 55 23–40. 10.1007/s00234-013-1231-0 23832006

[B80] Richards S. E. V. Van Hooser S. D. (2018). Neural architecture: From cells to circuits. *J. Neurophysiol.* 120 854–866. 10.1152/jn.00044.2018 29766767 PMC6139443

[B81] Ronchi S. Buccino A. P. Prack G. Kumar S. S. Schröter M. Fiscella M. (2021). Electrophysiological phenotype characterization of human iPSC-derived neuronal cell lines by means of high-density microelectrode arrays. *Adv. Biol.* 5:e2000223. 10.1002/adbi.202000223 33729694 PMC7610355

[B82] Rosenthal G. Váša F. Griffa A. Hagmann P. Amico E. Goñi J. (2018). Mapping higher-order relations between brain structure and function with embedded vector representations of connectomes. *Nat. Commun.* 9:2178. 10.1038/s41467-018-04614-w 29872218 PMC5988787

[B83] Saalfrank D. Konduri A. K. Latifi S. Habibey R. Golabchi A. Martiniuc A. V. (2015). Incubator-independent cell-culture perfusion platform for continuous long-term microelectrode array electrophysiology and time-lapse imaging. *R. Soc. Open Sci.* 2:150031. 10.1098/rsos.150031 26543581 PMC4632545

[B84] Sasaki T. Suzuki I. Yokoi R. Sato K. Ikegaya Y. (2019). Synchronous spike patterns in differently mixed cultures of human iPSC-derived glutamatergic and GABAergic neurons. *Biochem. Biophys. Res. Commun.* 513 300–305. 10.1016/j.bbrc.2019.03.161 30954214

[B85] Sauter E. J. Kutsche L. K. Klapper S. D. Busskamp V. (2019). “Induced neurons for the study of neurodegenerative and neurodevelopmental disorders,” in *Methods in molecular biology*, eds Ben-Yosef D. Mayshar Y. (New York, NY: Springer), 101–121. 10.1007/978-1-4939-9080-1_9 30900179

[B86] Schmieder F. Habibey R. Busskamp V. Büttner L. Czarske J. W. (2020). “Investigation of in vitro human iPSC-derived neuronal networks using holographic stimulation (conference presentation),” in *Proceedings of the optogenetics and optical manipulation* (Bellingham: SPIE). 10.1117/12.2546288

[B87] Schmieder F. Habibey R. Striebel J. Büttner L. Czarske J. Busskamp V. (2022). Tracking connectivity maps in human stem cell-derived neuronal networks by holographic optogenetics. *Life Sci. Alliance* 5:e202101268. 10.26508/lsa.202101268 35418473 PMC9008225

[B88] Segev R. Benveniste M. Shapira Y. Ben-Jacob E. (2003). Formation of electrically active clusterized neural networks. *Phys. Rev. Lett.* 90:168101. 10.1103/PhysRevLett.90.168101 12732015

[B89] Shi Y. Inoue H. Wu J. C. Yamanaka S. (2017). Induced pluripotent stem cell technology: A decade of progress. *Nat. Rev. Drug Discov.* 16 115–130. 10.1038/nrd.2016.245 27980341 PMC6416143

[B90] Silbereis J. C. Pochareddy S. Zhu Y. Li M. Sestan N. (2016). The cellular and molecular landscapes of the developing human central nervous system. *Neuron* 89 248–268. 10.1016/j.neuron.2015.12.008 26796689 PMC4959909

[B91] Sporns O. (2013). Making sense of brain network data. *Nat. Methods* 10 491–493. 10.1038/nmeth.2485 23722207

[B92] Steidl E. M. Neveu E. Bertrand D. Buisson B. (2006). The adult rat hippocampal slice revisited with multi-electrode arrays. *Brain Res.* 1096 70–84. 10.1016/j.brainres.2006.04.034 16716268

[B93] Stiles J. Jernigan T. L. (2010). The basics of brain development. *Neuropsychol. Rev.* 20 327–348. 10.1007/s11065-010-9148-4 21042938 PMC2989000

[B94] Taga A. Dastgheyb R. Habela C. Joseph J. Richard J. P. Gross S. K. (2019). Role of human-induced pluripotent stem cell-derived spinal cord astrocytes in the functional maturation of motor neurons in a multielectrode array system. *Stem Cells Transl. Med.* 8 1272–1285. 10.1002/sctm.19-0147 31631575 PMC6877769

[B95] Tau G. Z. Peterson B. S. (2010). Normal development of brain circuits. *Neuropsychopharmacology* 35 147–168. 10.1038/npp.2009.115 19794405 PMC3055433

[B96] Teppola H. Aćimović J. Linne M.-L. (2019). Unique features of network bursts emerge from the complex interplay of excitatory and inhibitory receptors in rat neocortical networks. *Front. Cell Neurosci.* 13:377. 10.3389/fncel.2019.00377 31555093 PMC6742722

[B97] Tibau E. Ludl A. A. Rudiger S. Orlandi J. G. Soriano J. (2020). Neuronal spatial arrangement shapes effective connectivity traits of in vitro cortical networks. *IEEE Trans. Netw. Sci. Eng.* 7 435–448. 10.1109/TNSE.2018.2862919

[B98] van Atteveldt N. Vandermosten M. Weeda W. Bonte M. (2021). How to capture developmental brain dynamics: Gaps and solutions. *NPJ Sci. Learn.* 6:10. 10.1038/s41539-021-00088-6 33941785 PMC8093270

[B99] Wang B. Ke W. Guang J. Chen G. Yin L. Deng S. (2016). Firing frequency maxima of fast-spiking neurons in human, monkey, and mouse neocortex. *Front. Cell Neurosci.* 10:239. 10.3389/fncel.2016.00239 27803650 PMC5067378

[B100] Wang I. E. Clandinin T. R. (2016). The influence of wiring economy on nervous system evolution. *Curr. Biol.* 26 R1101–R1108. 10.1016/j.cub.2016.08.053 27780051

[B101] Warm D. Schroer J. Sinning A. (2022). Gabaergic interneurons in early brain development: Conducting and orchestrated by cortical network activity. *Front. Mol. Neurosci.* 14:807969. 10.3389/fnmol.2021.807969 35046773 PMC8763242

[B102] Wilk N. Habibey R. Golabchi A. Latifi S. Ingebrandt S. Blau A. (2016). Selective comparison of gelling agents as neural cell culture matrices for long-term microelectrode array electrophysiology. *OCL* 23:D117. 10.1051/ocl/2015068

[B103] Yamamoto H. Moriya S. Ide K. Hayakawa T. Akima H. Sato S. (2018). Impact of modular organization on dynamical richness in cortical networks. *Sci. Adv.* 4:eaau4914. 10.1126/sciadv.aau4914 30443598 PMC6235526

[B104] Yuan X. Schröter M. Obien M. E. J. Fiscella M. Gong W. Kikuchi T. (2020). Versatile live-cell activity analysis platform for characterization of neuronal dynamics at single-cell and network level. *Nat. Commun.* 11:4854. 10.1038/s41467-020-18620-4 32978383 PMC7519655

[B105] Zafeiriou M.-P. Bao G. Hudson J. Halder R. Blenkle A. Schreiber M.-K. (2020). Developmental GABA polarity switch and neuronal plasticity in bioengineered neuronal organoids. *Nat. Commun.* 11:3791. 10.1038/s41467-020-17521-w 32728089 PMC7391775

[B106] Zeldenrust F. Wadman W. J. Englitz B. (2018). Neural coding with bursts—Current state and future perspectives. *Front. Comput. Neurosci.* 12:48. 10.3389/fncom.2018.00048 30034330 PMC6043860

[B107] Zhang Y. Pak C. H. Han Y. Ahlenius H. Zhang Z. Chanda S. (2013). Rapid single-step induction of functional neurons from human pluripotent stem cells. *Neuron* 78 785–798. 10.1016/j.neuron.2013.05.029 23764284 PMC3751803

