# Supplementary material for: Long-term morphological and functional dynamics of human stem cell-derived neuronal networks on high-density micro-electrode arrays
Source: Front Neurosci. 2022 Oct 4;16:951964. doi: 10.3389/fnins.2022.951964 (PMC9578684; doi:10.3389/fnins.2022.951964)
Supplement: Supplementary file 7 [file Data_Sheet_1.pdf]

frontiers

Supplementary Material

Long-term morphological and functional dynamics of human stem-cell-derived neuronal networks on high-density MEAs

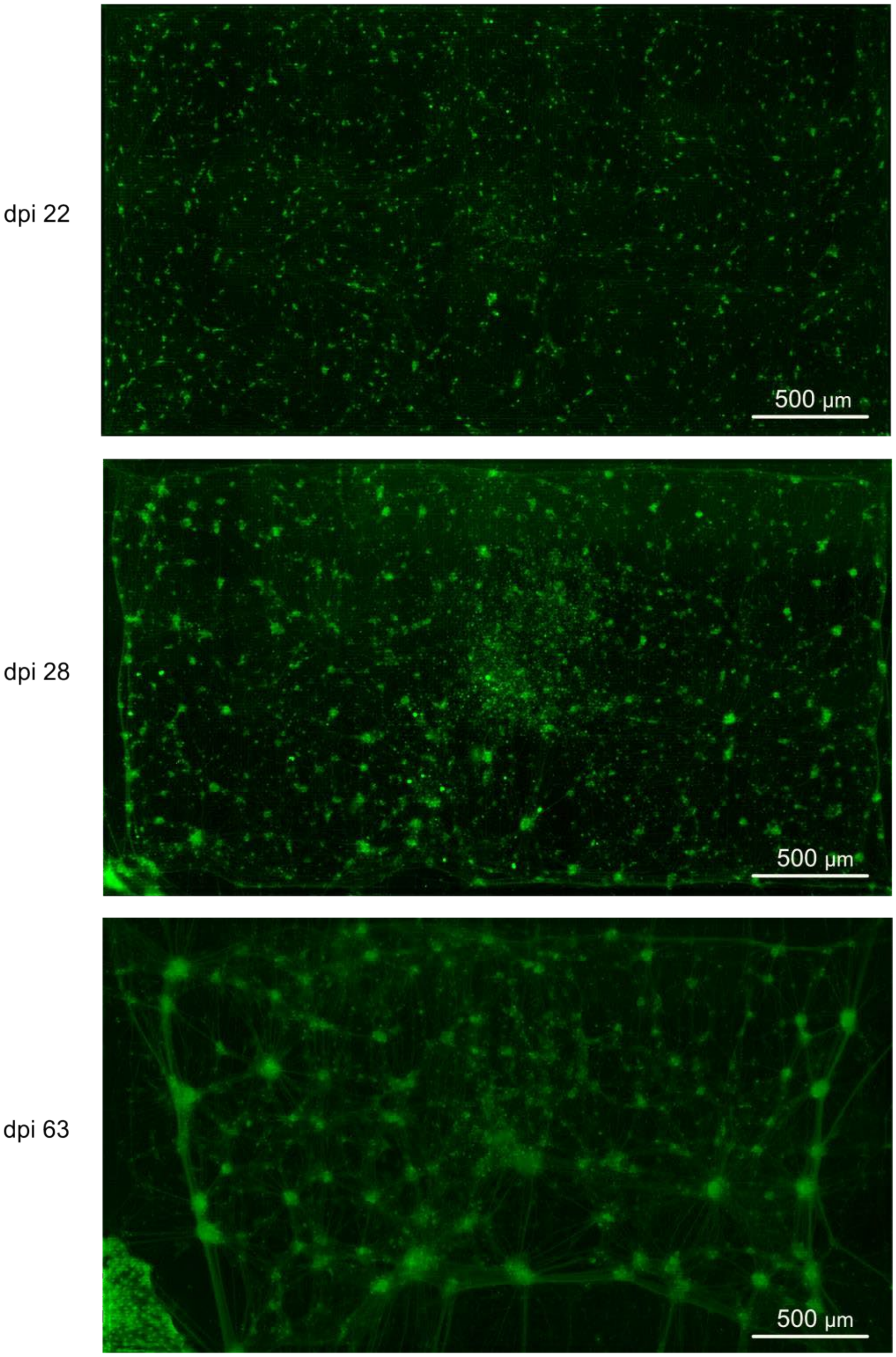

Supplementary Figure 1 Network morphology changes from homogenously distributed neurons to highly clustered circuits. At 22 dpi neurons are individually distributed across the sensor area. At 28 dpi neurons start forming small clusters and at 63 dpi large clusters of neurons have been formed by integration of small clusters and neuronal cell bodies.

dpi 63

dpi 63

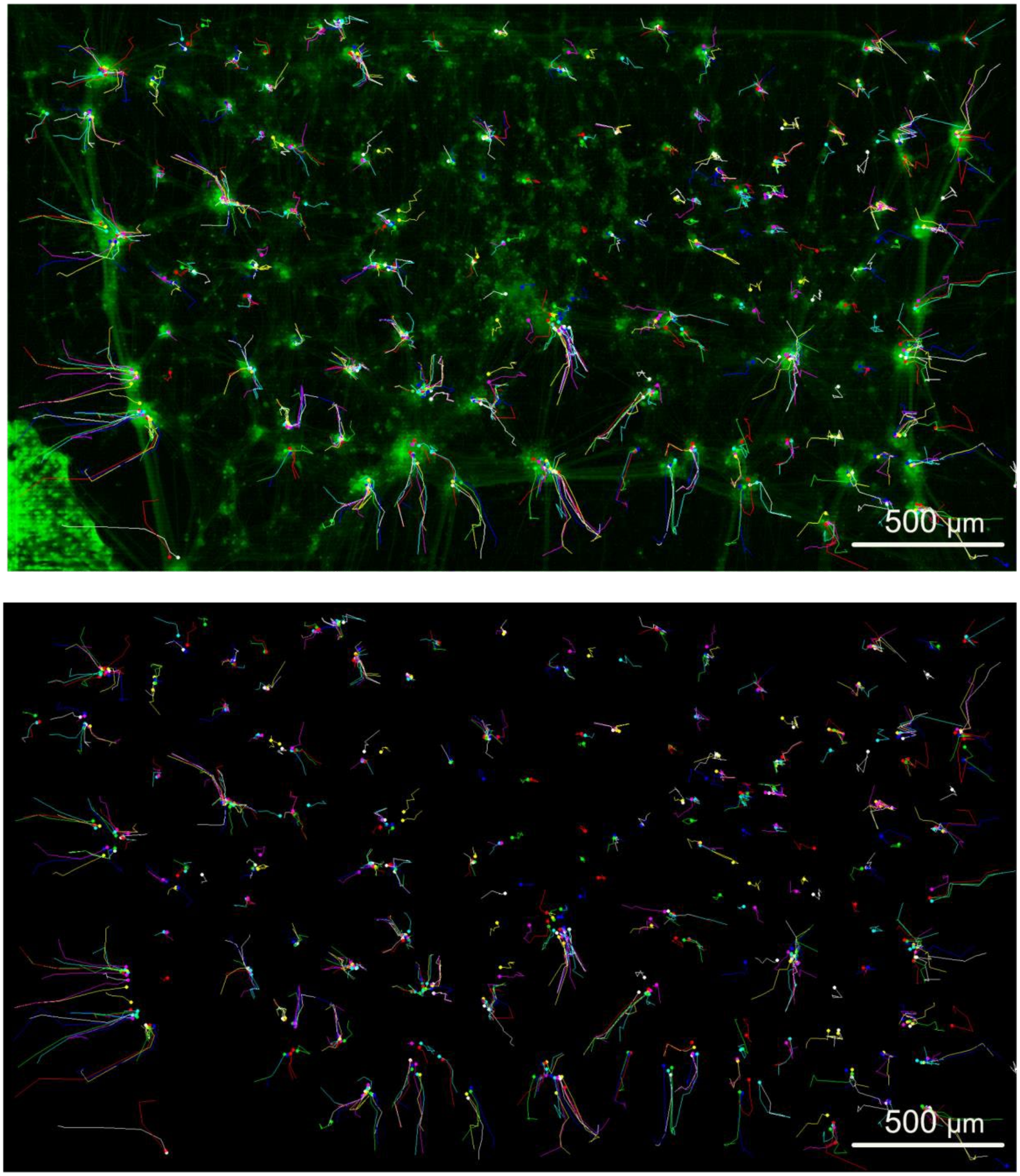

Supplementary Figure 2 Overlapped images of neuronal network morphology and trajectories of neuronal cell movement at 63 dpi. Tracking of 721 neurons was performed using a manual tracking plugin in ImageJ. Lower panel represents the trajectories only (no overlay) across the network at 63 dpi.

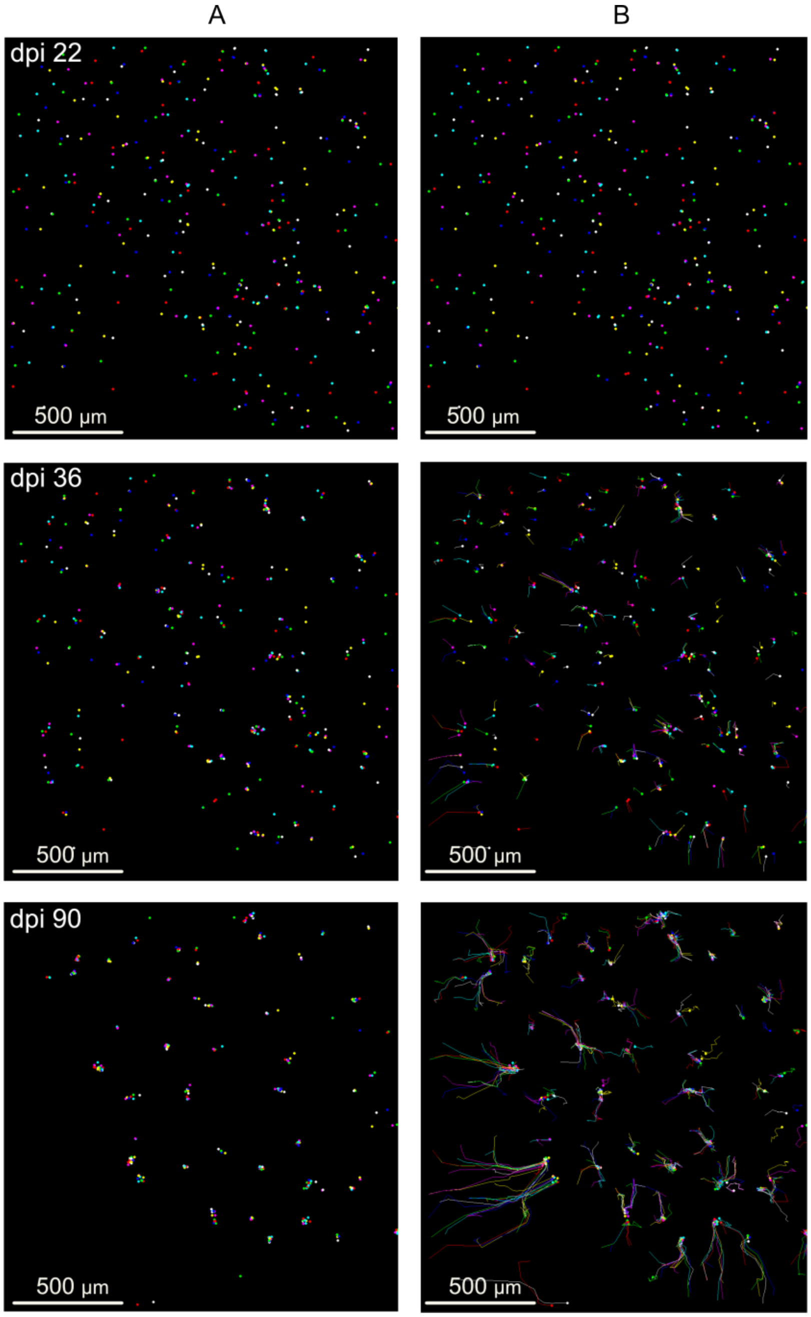

Supplementary Figure 3 Tracking neuronal cell body clusterization and trajectories of neuronal cell movements of MEA 1. Images illustrate a half of the sensor area at 22, 36 and 90 dpi. A) Neurons are marked with colored dots. Formation of neuronal clusters can be observed over time. B) Trajectory of neuronal cell migration during the network development.

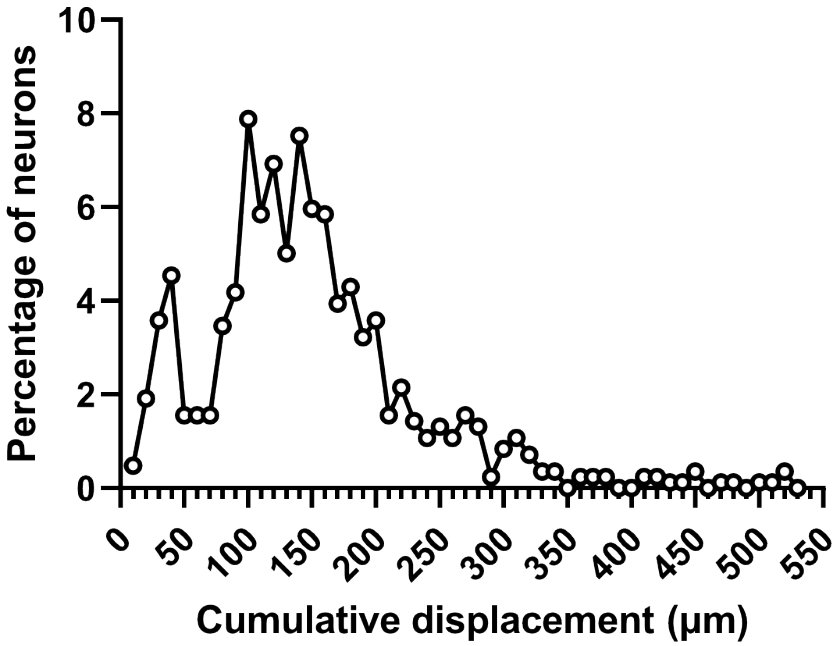

Supplementary Figure 4 Distribution of neuronal cell displacement in two months. Data have been collected from 904 individual neurons in 3 HD-MEAs between 28 dpi and 76 dpi. Overall shift of each neuron during this time was calculated based on cumulative displacement of that neuron. Data has been represented in 10 µm intervals. Neurons showing more than 550 µm displacement (less than 2% of all tracked neurons) are not shown in this graph.

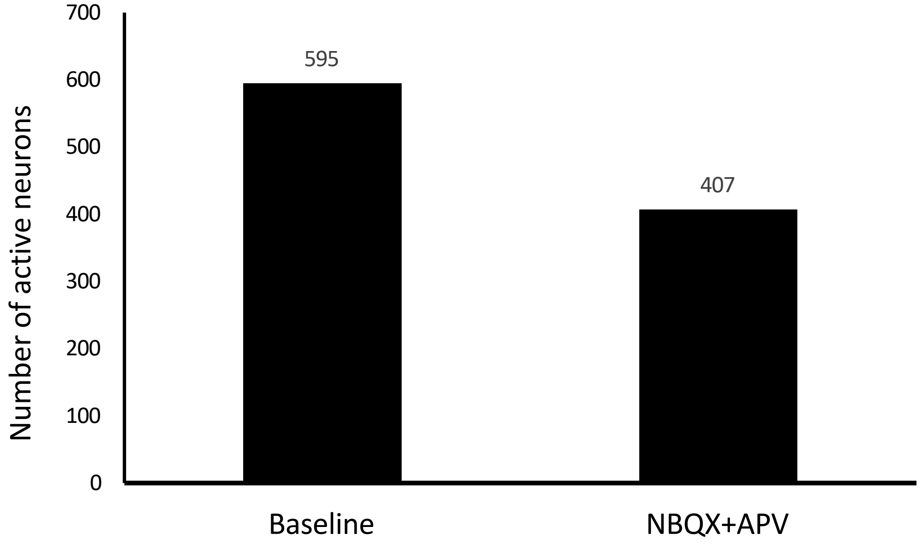

Supplementary Figure 5 Number of active neurons in baseline and NBQX+APV treated condition. Data collected from 2 MEAs used in the experiment. Neurons were considered active if they showed more than 0.2 Hz firing rate.

Videos:

Supplementary Video 1 Time lapse video of network morphology development. Each image frame contains 40 stitched images prepared from the entire sensor area.

Supplementary Video 2 Time lapse video of neuronal cell movement, clusterization and trajectories of movement. Neuronal cells were individually tracked based on whole network morphology images at different days. A) Shows overlapped morphology and tracked neurons on different days for 3 months. B) Magnified view of top-left corner of the sensor area (marked by red in A). C) Illustrates the trajectories of neuronal cell migration and movement during the development. D) Represents tracked neuronal cell body movements and formation of neuronal clusters during the network development. (n=721 neurons, N=1 MEA).

Supplementary Video 3 Time lapse video of network activity image development. Each image frame represents the results of a full activity scan taken from the entire sensor area. Activity of each electrode was scanned for 30 seconds on each dpi. Approximately 25 minutes of recording was required in total to scan the whole sensor area. Firing rates have not been normalized across different days and color coding represents different scales of the activity between frames.

Supplementary Video 4 Time lapse video of a network morphology image (left) and overlapped morphology and activity images (right). Both morphology and activity images show a magnified area of the same MEA from 22 dpi to 76 dpi (same as in Figure 2D). Each yellow square covers approximately 7x7 electrodes of sensor area and the whole image contains around 3200 electrodes.

Supplementary Video 5 Time lapse video of iNGN-ChR2 network morphology developing on a standard MEA with 64 electrodes. iNGN cells are expressing Channelrhodopsin-2 (ChR2) tagged with YFP. Each image frame contains 20 stitched images covering the whole sensor area (1.4 mm^2). This time-lapse video is prepared from previously published data (Schmieder et al., 2022).

Supplementary Video 6 Time lapse video of electrode raster plots during network development. Each row represents 20 seconds activity in one selected electrode and each point represents one recorded action potential. Color codes represent different regions of the sensor area.

References

Schmieder, F., Habibey, R., Striebel, J., Büttner, L., Czarske, J., and Busskamp, V. (2022). Tracking connectivity maps in human stem cell–derived neuronal networks by holographic optogenetics. *Life Sci. Alliance* 5, e202101268. doi:10.26508/lsa.202101268.
